# Accounting for detection probability with overestimation by integrating double monitoring programs over 40 years

**DOI:** 10.1371/journal.pone.0265730

**Published:** 2022-03-25

**Authors:** David Vallecillo, Matthieu Guillemain, Matthieu Authier, Colin Bouchard, Damien Cohez, Emmanuel Vialet, Grégoire Massez, Philippe Vandewalle, Jocelyn Champagnon

**Affiliations:** 1 Tour du Valat, Research Institute for the Conservation of Mediterranean Wetlands, Le Sambuc, Arles, France; 2 OFB, Unité Avifaune migratrice, La Tour du Valat, Le Sambuc, Arles, France; 3 Observatoire Pelagis, UMS 3462 CNRS-LRUniv ADERA, La Rochelle, France; 4 UMR Ecobiop, e2S, Université de Pau et Pays de l’Adour, INRAE, Saint-Pée sur Nivelle, France; 5 Parc Naturel Régional de Camargue, Mas du Pont de Rousty, Arles, France; 6 Les Amis des Marais du Vigueirat, Chemin de l’Etourneau, Mas-Thibert, France; 7 SNPN-RNN de Camargue, Arles, France; USDA Forest Service, UNITED STATES

## Abstract

In the context of wildlife population declines, increasing computer power over the last 20 years allowed wildlife managers to apply advanced statistical techniques that has improved population size estimates. However, respecting the assumptions of the models that consider the probability of detection, such as *N*-mixture models, requires the implementation of a rigorous monitoring protocol with several replicate survey occasions and no double counting that are hardly adaptable to field conditions. When the logistical, economic and ecological constraints are too strong to meet model assumptions, it may be possible to combine data from independent surveys into the modelling framework in order to understand population dynamics more reliably. Here, we present a state-space model with an error process modelled on the log scale to evaluate wintering waterfowl numbers in the Camargue, southern France, while taking a conditional probability of detection into consideration. Conditional probability of detection corresponds to estimation of a detection probability index, which is not a true probability of detection, but rather conditional on the difference to a particular baseline. The large number of sites (wetlands within the Camargue delta) and years monitored (44) provide significant information to combine both terrestrial and aerial surveys (which constituted spatially and temporally replicated counts) to estimate a conditional probability of detection, while accounting for false-positive counting errors and changes in observers over the study period. The model estimates abundance indices of wintering Common Teal, Mallard and Common Coot, all species abundant in the area. We found that raw counts were underestimated compared to the predicted population size. The model-based data integration approach as described here seems like a promising solution that takes advantage of as much as possible of the data collected from several methods when the logistic constraints do not allow the implementation of a permanent monitoring and analysis protocol that takes into account the detectability of individuals.

## 1. Introduction

While wildlife populations decline across multiple taxa, population dynamics help appreciating temporal variability in population size and the subsequent risk of collapse [1]. Over the last 20 years, increased computing power allowed wildlife managers to apply advanced statistical techniques previously developed theoretically, which has improved population size estimates.

Sources of error such as imperfect detection [2, 3], biased abilities to count animals that are detected [4–7], and misidentification of species [8, 9] can introduce considerable estimation bias [10] and reduce the survey’s power to detect trends [6, 11–13]. Counts may provide reliable information about population trends if detection probability remains constant over time [5, 14], yet such a situation is rarely met in practice [13]. Observers in long-term surveys often change annually or gain experience through time [15], both of which affect detection [16].

Several survey designs and modelling approaches exist to account for and correct imperfect detection in studies of population dynamics. For instance, distance sampling [17], double sampling [18] and double-observer [19] approaches are widely used techniques to estimate abundance of wild animal populations. At the ends of the 1990s, state-space models have been developed to deal with the observation error and the latent ecological process of interest simultaneously [20].

More specifically, a special case of the state-space models, binomial mixture models (*N*-mixture, [21]) jointly estimates local abundance and detection probability with a metapopulation design and counts without individual identification or distance measurements [22–25]. Although promising and powerful, the assumptions of *N*-mixture models are strong and even small violations can lead to large estimation biases [26, 27]. Respecting the assumptions of *N*-mixture models requires the implementation of a rigorous and demanding monitoring protocol that may prove too stringent in some field conditions. False-positive counting errors (double-counting and misidentification) can be prominent in the observation process. Although a steadily increasing number of papers have dealt with false-positive errors [9, 28, 29], it remains difficult to collect all the necessary data to account for them. The binomial distribution used in the *N*-mixture assumes that the modelling of the observation process is actually dominated by false-negative errors, whereas false-positive errors are rare or absent. However, this assumption can be problematic in the case of gregarious species, which are common in the animal kingdom. This is particularly the case when counting flocks of animals such as wintering waterfowl, which is not an easy task owing to their high gregariousness, mixing of species, and harsh weather conditions [30, 31]. In addition, monitoring may lead to a violation of the population closure assumption when disturbance during a survey causes the displacement of individuals.

Joint analysis of several datasets from heterogeneous protocols is increasingly popular because it takes advantage of as much possible of data collected [32–35]. Model-based data integration is particularly relevant to estimating species’ distributions [36] or population dynamics [37]. For instance, some studies relaxed some assumptions behind *N*-mixture models and estimated abundance of open populations using multiple sources of data, including telemetry data on locations of individuals [38]. *N*-mixture models aim to estimate absolute abundance. However, a reliable index of abundance (relative abundance) is often enough to reveal and tackle changes in relative population size, and plan conservation actions when needed.

Here we developed a state-space model to evaluate wintering waterfowl numbers in the Camargue, southern France, where both ground and aerial censuses have been performed for decades. We have taken into consideration a conditional probability of detection which corresponds to estimation of probability detection indices that depend on the value set for one of the parameters. The overall high probability of detection of waterfowl during the winter, because of their gregarious habits, the large number of sites (wetlands within the Camargue delta) and years monitored made it possible to combine both terrestrial and aerial surveys (which constituted spatially and temporally replicated counts; [42]), while taking into account changes in observers over the study period. Such an approach, while more *ad hoc* than *N*-mixture models, allowed us nevertheless to integrate several datasets, which have undergone changes in protocol over time (but crucially not at the same time), to accurately assess population dynamics.

## 2. Methods

### 2.1 Study area and species

The Camargue is a wide delta (~ 150 000 ha) at the mouth of the Rhône River (southern France) to the Mediterranean Sea. The delta is composed of over 60,000 ha of wetlands and 30,000 ha of protected areas; the remaining area is mostly farmland. Since monitoring began in the 1960s, regular wintering bird counts have shown the international importance of the Camargue (*sensu* the Ramsar Convention) for many species including Common Teal (*Anas crecca*), Mallard (*Anas platyrhynchos*) and Common Coot (*Fulica atra*). The counts show that the Camargue has hosted on average 25 900 Teal ± 11 200 SD, 25 100 Mallard ± 13 300 SD and 21 300 Common Coot ± 8 100 SD in January (month used by Wetlands International for the International Waterbird Census) from 1976 to 2020. These three species are also the most abundant species in the Camargue waterfowl community: Common Teal, Mallard and Common Coot account on average for 28.7% (± 11.8 SD, n = 43 years), 30.5% (± 15.6 SD, n = 43 years) and 25.5% (± 16.9 SD, n = 43 years) of the total number of *Anatidae* + *Rallidae* during mid-winter, respectively. During the day, these species congregate in high concentrations on marshes and ponds, called day-roosts, which are generally extensive and open [39]. Ducks (e.g., Common Teal and Mallard) generally allocate little time to feeding in these areas, around a quarter of the day or less because they feed actively during the night, while the Common Coot has mainly diurnal feeding activity [39].

### 2.2 Population counts

**Fig 1:**
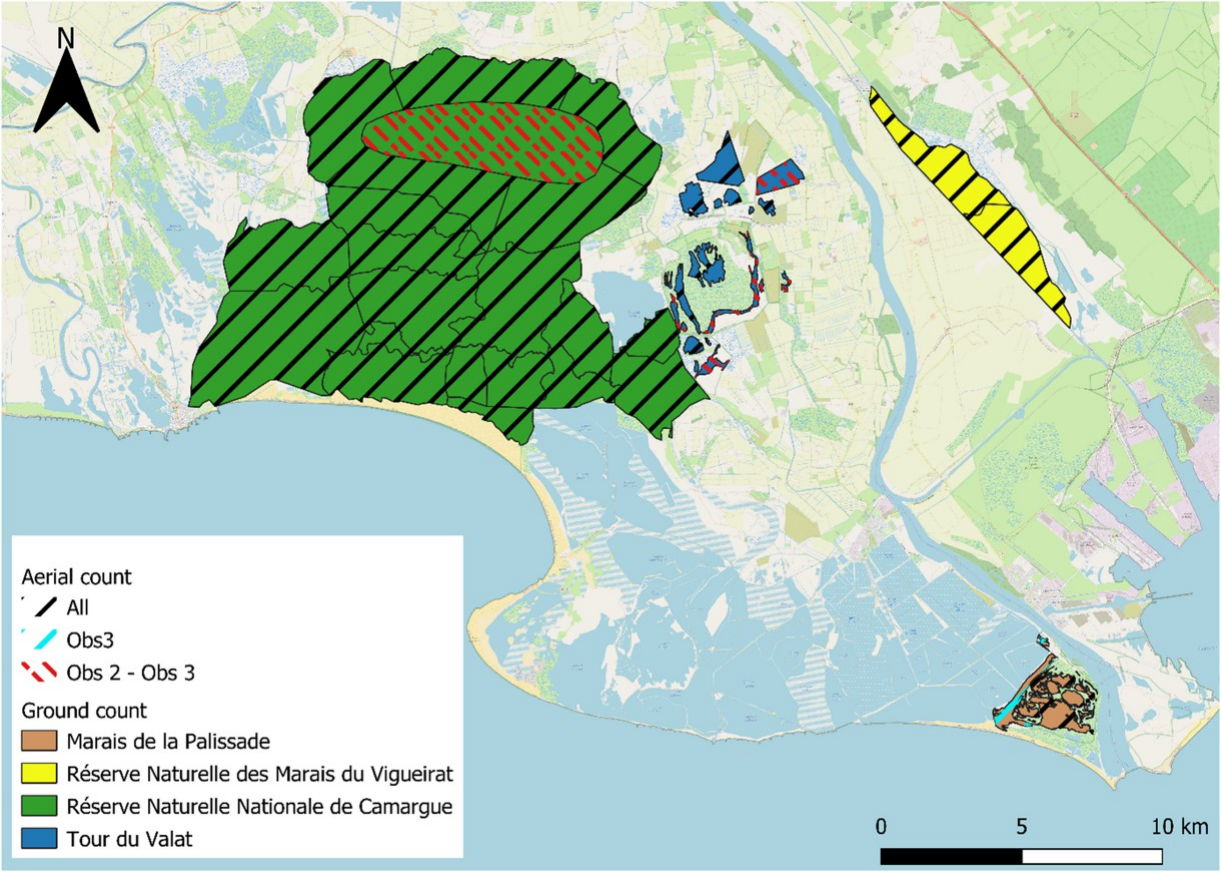
Location of the 40 sites in the study area. Ten sites were distributed within the Tour du Valat (in blue), 21 sites at the Réserve Naturelle Nationale de Camargue (in green), two sites at the Réserve Naturelle des Marais du Vigueirat (in yellow) and seven sites at the Marais de la Palissade (in brown). For aerial counts, 32 sites were counted by the first observer (1975–2002), 39 sites by the second (2004–2013) and 40 sites by the last (2014–2020). Ground data were collected by different observers and the year in which monitoring began differed among sites. Background map is provided by © OpenStreetMap contributors available under the Open Database License (https://www.openstreetmap.org/copyright).

The Camargue has been entirely covered by aerial monitoring, which includes 130 polygons. We focused only on the 40 with overlap with the ground counts. The choice of the number of polygons used was restricted by the number of these having been monitored for a long time from the ground. The study area covered by the aerial and ground counts used in this study represents a total of 18,333 ha and the polygons range in size from 9 to 1,885 ha (Fig 1). These polygons correspond to the visual delimitation of coherent water bodies making the spatialization of the counts possible. The term *site* will hereafter be used to refer to a polygon of the study area with an associated count per species.

Waterbirds are counted in these 40 sites by plane and from the ground, monthly from September to March every year since 1964, with only 35 missing values in total for aerial monitoring (ranging from eight missing years in March to only one in January). The first ten years were excluded due to changes in protocol (methodology, division of counting sectors, etc.). Each aerial count was carried out as close as possible to the 15^th^ of the month, generally on a Tuesday to reduce disturbance that weekend hunting could induce on the distribution of waterbirds [39]. The counting date was shifted when weather conditions were too adverse (low clouds, heavy wind, fog). Over the whole area, a similar track which made it possible to fly over all the sites (6 hours flight) was followed each month to count the birds. When a group was detected and to enable the identification of birds, the pilot flew towards the group and forced them to take flight (by circling over a given wetland). This counting method was based on easily detectable diurnal species (gregarious species), applied to a relatively limited geographical area and almost all the day-roosts used in the delta by the species to be counted were visited; the method is considered exhaustive in its spatial coverage and differs from a transect methodology [39]. The plane flew at a low altitude (about 60 to 80 m) at a speed of about 180 km/h. Bird counts were recorded on a voice recorder by an observer sitting next to the pilot, distinguishing the number of individuals per species per wetland site [39]. Observers recorded the number of individuals of each of the 14 most common waterbird species present on each pond (Common Coot, Mallard, Northern Shoveler, Common Teal, Eurasian Wigeon, Gadwall, Common Pochard, Tufted Duck, Red-crested Pochard, Northern Pintail, Greylag Goose, Mute Swan, Tundra Swan, Common Shelduck) and the pilot did not participate in the observations. Sex composition was not considered. A pond generally corresponded to a site. Only three observers recorded bird numbers since the beginning of the counts: Observer 1 (Obs1) from 1964 to 2001, Observer 2 (Obs2) from 2003 to 2012 and Observer 3 (Obs3) since 2013, increasing reliability of the surveys over the time [6]. Each observer trained his successor, in turn. The number of sites monitored has increased over time. Thirty-two sites were counted by the first observer (1975–2002), 39 sites by the second (2004–2013) and 40 sites by the last (2014–2020), in order to consider newly appearing day-roosts (Fig 1).

In parallel, ground counts were carried out on the 40 sites on the same day or within a few days of aerial counts (on average 3.3 days ± 3.3 SD). Ground data were collected by different observers (identity of observers not recorded) and were initiated at different dates in the various sites. Several of the monitoring sites are protected areas (albeit with different protection statuses): The Tour du Valat (10 sites, started in 1979 with 40% of missing values reflecting months during the entire study period without ground surveys at the Tour du Valat sites), the Réserve Naturelle Nationale de Camargue (21 sites, started in 1989, 41% of missing values), the Réserve Naturelle des Marais du Vigueirat (2 sites, started in 1989, 46% of missing values), and the Marais de la Palissade (7 sites, started in 1992, 58% of missing values). Two methods of counting from the ground have been set up according to the protected areas and characteristics of their environment. Depending on the protected area, the observers either counted from a vantage point without disturbing the birds or conversely moved and flushed the birds away to count them when flying. For the Réserve Naturelle Nationale de Camargue the observers counted the birds from a fixed point and for the Marais de la Palissade and the Marais du Vigueirat they moved and flushed the birds away to count them. For the Tour du Valat a change of method occurred from fixed point counting at the beginning of the surveys to voluntary disturbance from 2006 onwards. In the same way as aerial counts, ground observers recorded the number of individuals of each waterbird species in each site without considering sex.

### 2.3 Statistical analyses

The count data from the 40 sites over up to 44 years were analysed with state-space models [40, 41] using a Bayesian framework [42]. State-space models consist of a set of linked models: (1) a model for the state process, (2) a model for the observation process and (3) a model for the initial state. The state process describes the dynamics of the state (in our case the bird abundance index) over time and for each site, and the observation model links the observations (counts) to the state. The model takes into account random sampling errors (counting errors) from both sources of data [42]. The model represents environmental stochasticity as log-Student process noise. We assumed a stochastic exponential population growth in an unlimited environment (with no density-dependence) for the dynamic of the state because it is expected that the carrying capacity of the sites is not reached [39]. In our case, the modelling process estimates an abundance index corrected by a conditional probability of detection (see below). Thus, the abundance index *N* in a site *s* in year *t* is estimated by:

$$N_{s,t}=N_{s,t‐1}\times \exp(r_{s,t})$$ (1)

where *r*_s,t_ is the instantaneous growth rate of site *s* from year *t—1* to *t*. *N*_*s*,*t*_ is the population size for the month of September. The growth rate of site *s* and year *t* is modelled by a first order random walk given by:

$$r_{s,t}∼\mathrm{Normal}(r_{s,t‐1},\;\sigma_{\mathrm{year}}^{2})$$ (2)

where the growth rate in year *t* depends on the growth rate in year *t-1*. The growth rate is a normally distributed random effect whose variance $\sigma_{\mathrm{year}}^{2}$ is the variability of the growth rates among years whatever the site. We expected some temporal autocorrelation between growth rates due to intrinsic causes in the population dynamics of waterfowl population size [43]. We therefore considered that the growth rate from the previous year *t-1* should partly explain the growth rate in the current year *t*. To define the initial state (population size in the year when the monitoring started) we estimated the size of the population for each site *s* in the first year as:

$$\log{(N}_{s,1})∼\mathrm{Normal}(\mathrm{mu},\;\sigma_{\mathrm{site}}^{2})$$ (3)

where *mu* is the expected abundance during year 1975 and $\sigma_{\mathrm{site}}^{2}$ is the variability of the size of the population among sites.

The model contained a log-Student observation error. Student’s t-distribution with a low degree of freedom was chosen for the observation process to take into account the major counting error that may occur occasionally when an observer does not detect a large group of ducks. In addition, this parametrisation serves to buffer the large variation in abundance due to extreme weather conditions. Thus, counts for each method *i*, each month *m*, each site *s* in year *t* are modelled as:

$${\log\_\mathrm{Count}}_{i,m,s,t}∼\mathrm{Student}(\log(N_{s,t})+\alpha_{m,t}+\log(p_{i,s,t}),\sigma_{i}^{2},\;\nu )$$ (4)

where *N*_*s*,*t*_ is the abundance index, *α*_*m*,*t*_ is the seasonal pattern, *p*_*i*,*s*,*t*_ is a detection probability, $\sigma_{i}^{2}$ are the specific counting method residual errors and ν are the degrees of freedom set at 4 [44]. The seasonal pattern *α*_*m*,*t*_ has been hierarchically decomposed with an interannual stochasticity $\sigma_{\mathrm{month}2}^{2}$ in the month effect *δ*_*m*_ as:

$$\alpha_{m,t}∼\mathrm{Normal}(\delta_{m},\;\sigma_{\mathrm{month}2}^{2})$$ (5)

where *δ*_*m*_ are the expected values of the month effect for the month *m* and $\sigma_{\mathrm{month}2}^{2}$ is the interannual variability of the seasonal pattern. *δ*_*m*_, the month effect depending on the effect of the previous month (*δ*_*m-1*_), is modelled by a first order random walk given by:

$$\delta_{m}∼\mathrm{Normal}(\delta_{m‐1},\;\sigma_{\mathrm{month}1}^{2})$$ (6)

where $\sigma_{\mathrm{month}1}^{2}$ is the intermonthly variability. *δ*_*1*_, the month effect for September, is set to 0 for identifiability. In this way, we take into account the bell-shaped seasonal abundance pattern observed in the data (with parameter *δ*_*m*_) while also accounting for the interannual variations of this pattern (with parameter *α*_*m*,*t*_). Indeed, ducks are gregarious and quite site-faithful so that newcomers join during the winter to already existing flocks on diurnal resting sites [45, 46].

The variations in the conditional probability of detection has been modelled as a function of protected areas (for ground counts) and observers (for aerial counts) using a logit-linear model to *p*_*i*,*s*,*t*_:

$$p_{i,s,t}=\mathrm{inv}\_\mathrm{logit}(\beta_{i}+\gamma_{i,s,t})$$ (7)

with the counting method effect *β*_i_ (i = 1 for the aerial method which corresponds to the value of conditional probability of detection for Obs1 and i = 2 for the ground method which corresponds to the value of conditional probability of detection for Réserve Naturelle des Marais du Vigueirat) and *γ*_*i*,*s*,*t*_ takes into account intra-method variations (Tables 1 and 2). Two values were estimated for the parameter *γ*_*1*,*s*,*t*_, which correspond to the two changes of observers during aerial counts (Table 1). Four values were estimated for the parameter *γ*_*2*,*s*,*t*_ which correspond to the three protected areas (Tour du Valat, Réserve Naturelle Nationale de Camargue, Marais de la Palissade) with a temporal change in the counting method at the Tour du Valat due to the change in the protocol in 2006 from undisturbed count from a distance to count with disturbance (Table 2). Considering a protected areas effect (grouping of sites) allowed us to consider mainly the differences in detection between the ground counting methods used in each protected area. We assumed no site effect in the detection probability for aerial counts as the same observer covered all sites by using the same method. Even though it is unlikely to have a constant site effect over such a long period, we assume that this part of the variability is represented by the residual error. No observer effect was considered for ground counts because the identity of the observers was not recorded.

**Table 1:** Parametrization of the linear predictor of the conditional detection probability for aerial counts.

Aerial counts *p*_*1*,*s*,*t*_	t = 1, …,27	t_1_ = 28, …,37	t_2_ = 38, …,44
s = 1, …, 40	Obs 1	Obs 2	Obs 3
	*β*_1_	$\beta_{1}+\gamma_{1,s,t_{1}}$	$\beta_{1}+\gamma_{1,s,t_{2}}$

**Index *s* corresponds to sites and index *t* to years**. Three values of parameters were estimated for aerial counts (one for *β* and two for *γ*).

**Table 2:** Parametrization of the linear predictor of the conditional detection probability for ground counts.

Ground counts *p*_*2*,*s*,*t*_	t = 1, …, 44	
	t_1_ = 1, …, 31	t_2_ = 32, …, 44
s_1_ = 1, …, 7	Marais de la Palissade	
	$\beta_{2}+\gamma_{2,s_{1},t}$	
s_2_ = 8, …, 28	Réserve Naturelle Nationale de Camargue	
	$\beta_{2}+\gamma_{2,s_{2},t}$	
s_3_ = 29, …, 38	Tour du Valat	Tour du Valat new counting method
	$\beta_{2}+\gamma_{2,s_{3},t_{1}}$	$\beta_{2}+\gamma_{2,s_{3},t_{2}}$
s = 39, 40	Réserve Naturelle des Marais du Vigueirat	
	*β*_2_ = 0	

**Index *s* corresponds to sites and index *t* to years.** Four values were estimated for ground counts (four for *γ*). Parameter *β*_2_ was set to 0 for identifiability.

To overcome identifiability issues in parameter estimations, we arbitrarily set parameter *β*_2_ to 0 (which is equal to a probability of detection index of 0.5) which corresponds to the index of detection probability for the two sites of Réserve Naturelle des Marais du Vigueirat (Table 2). In this way we do not obtain an actual probability of detection but a conditional probability that allows us to correct the abundance index for shifts in the probability of detection. Yet, since we were interested in the change of population sizes rather than in their absolute values, this limitation does not pose a serious problem for our inference.

We specified weakly informative priors based on knowledge about the parameters adapted to our data (the priors are given in S1 Appendix) by conducting a Prior Predictive Check [47] (for the code see S1 Appendix). We used JAGS, a MCMC sampler [48, 49], to fit the model (for the code see S2 Appendix) that was run from R via package R2jags [49, 50]. A total of 40,000 samples were simulated from the posteriors with the first 1,500 as burning. We only retained every 10^th^ sample and used three chains with dispersed initial values to check the convergence of the simulations. Convergence was satisfactory (in all cases the Brooks–Gelman–Rubin criterion was $\hat{R}$ < 1.1; [51]). Before being applied to the data set, we measured estimation bias by conducting a sensitivity analysis of the model based on a simulated dataset (see S3 Appendix). To ensure that missing data did not bias the estimates of the detection parameters, the sensitivity analysis was performed with the same proportions of missing data as in the real dataset. A Bayesian fit analysis was performed to measure the fit of the model to the observed data (see S4 Appendix).

The results were compiled from the posterior distributions of the detection probability indices, for each of the aerial observers and protected areas, and the abundance index.

## 3. Results

The model estimated abundance indices for each species and site with conditional detection probabilities for each method (and observer in the case of aerial counts). The sensitivity analysis did not reveal biased model estimates in the differences in the probability of detection (conditional probability of detection) whatever the value set for the Réserve Naturelle des Marais du Vigueirat (range set from 0 to 1.4, for the sensitivity analysis see S3 Appendix).

**Fig 2:**
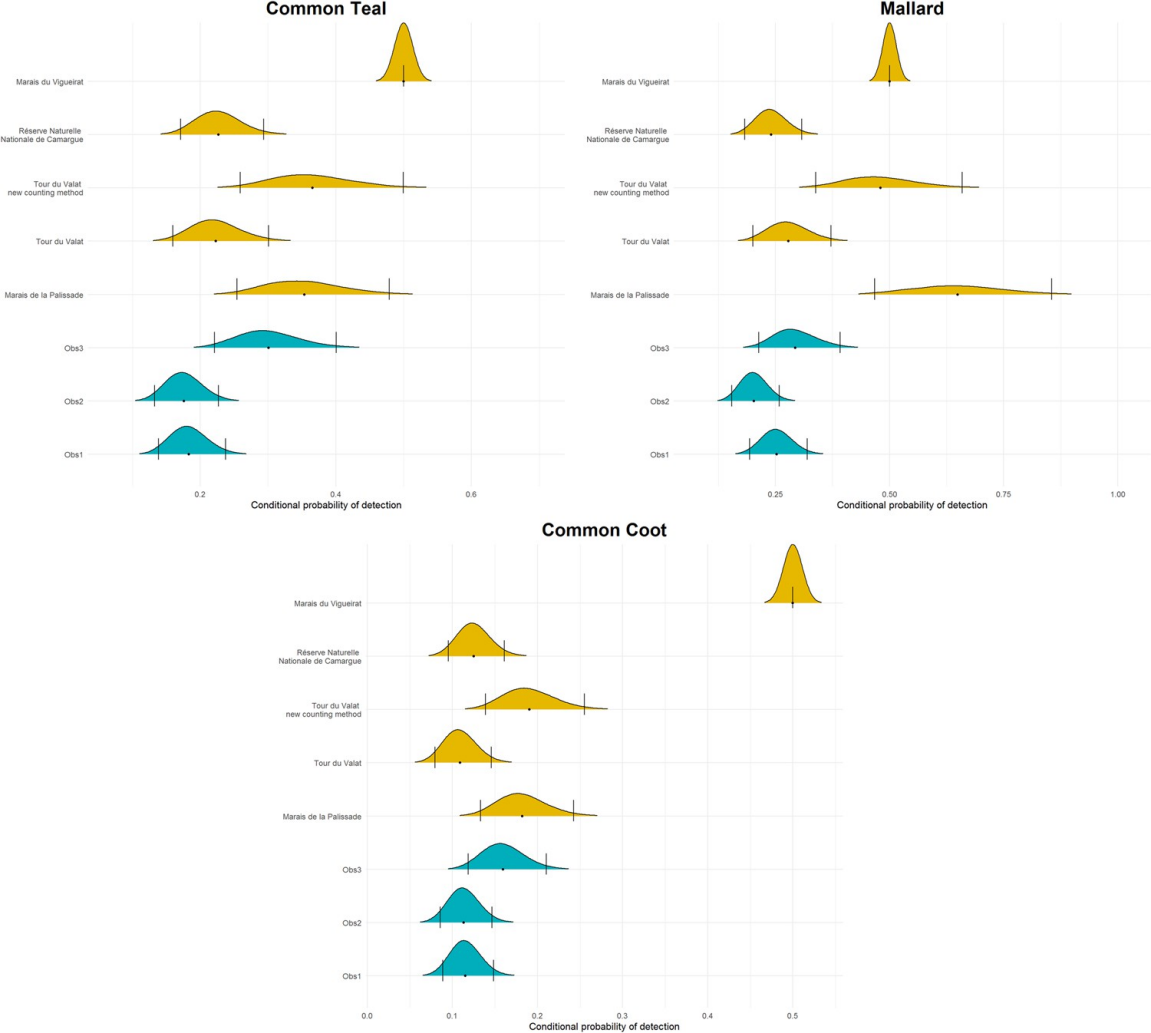
Density plot representing posterior distributions of the conditional detection probability for each of the observers (blue for aerial counts) and protected areas (yellow for ground counts). The dot represents the average of conditional probability of detection for each of these parameters and the dashed lines the associated 95% credible interval.

For the aerial counts and for all three species, Obs1 detected the same proportion of the number of individuals on average as Obs2, while Obs3 detected on average more individuals than Obs1 and Obs2, and hence had the highest index of the probability of detection (Fig 2). For Common Teal, Obs3 had a 93% probability of detecting more individuals than Obs1 (proportion of MCMC samples that were higher than the upper limit of Obs1’s credible interval) and a 96% probability of detecting more individuals than Obs2. On average, Obs3 detected 1.7 ± 0.2 SD times more individuals than Obs1 or Obs2 (Fig 2). For Mallard, Obs3 had a 27% probability of detecting more individuals than Obs1 and a 73% probability of detecting more individuals than Obs2. On average, Obs3 detected 1.2 ± 0.1 SD times more individuals than Obs1 and 1.4 ± 0.2 SD times more individuals than Obs2 (Fig 2). For Common Coot, Obs3 had a 67% probability of detecting more individuals than Obs1 and a 70% probability of detecting more individuals than Obs2. On average, Obs3 detected 1.4 ± 0.1 SD times more individuals than Obs1 or Obs2 (Fig 2).

All species considered, 1.6 ± 0.1 SD times as many individuals were detected from the ground (i.e., with and without flushing the birds) compared to aerial counts. 2.1 ± 0.3 SD times more birds were detected from the ground when the protocol had observers flushing the birds away to count them compared to aerial counts and ground counts without flushing the birds (the Marais de la Palissade, the Marais du Vigueirat and the Tour du Valat after the change in counting method, Fig 2).

**Fig 3:**
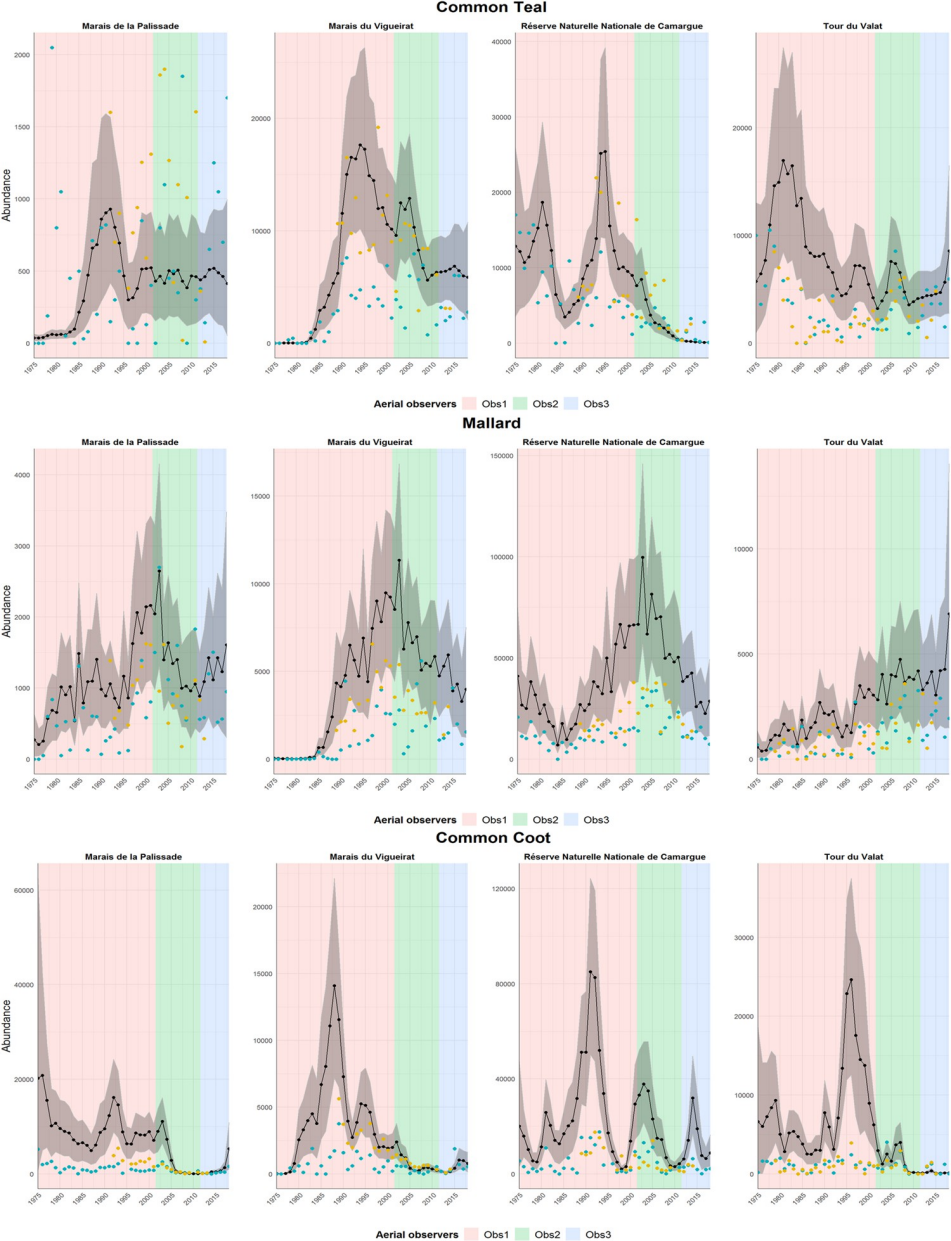
Estimation of the abundance index (black curve with its 95% credible intervals) over the entire monitoring period for the month of January and for each of the protected areas. Aerial counts are represented by blue dots. Ground counts are represented by yellow dots.

For all species, the abundance index values estimated by the model were in most cases higher than the bird numbers recorded during monitoring, i.e. bird counts underestimated actual bird numbers (Fig 3). However, the estimates of the abundance index did successfully capture the temporal dynamics observed in the count data (Fig 3). For instance, the peaks of abundance observed for Common Teal between 1990 and 1997 at the Marais du Vigueirat and the Réserve Naturelle Nationale de Camargue were well estimated by the model, as were the peaks of abundance observed between 2000 and 2008 at the Marais du Vigueirat and the Tour du Valat (Fig 3). Moreover, the abundance index was positively correlated with aerial and ground counts for the three species studied (Spearman’s test; Common Teal abundance index: aerial counts, *ρ* = 0.76, ground counts, *ρ* = 0.66; Mallard abundance index: aerial counts, *ρ* = 0.87, ground counts, *ρ* = 0.91; Common Coot abundance index: aerial counts, *ρ* = 0.72, ground counts, *ρ* = 0.74).

## 4. Discussion

We developed a state-space model to better assess population dynamics [25] of wintering waterfowl in the Camargue by estimating abundance indices based on two monitoring methods. The aim of this paper was not to have a demographic model estimating the population size and the underlying demographic processes at the scale of the Camargue (evaluation of the factors responsible for fluctuations in numbers) but to have a model that corrects the imperfections of detection in order to have a first step of better estimation of abundance by using several sources of data that are simultaneously available. The estimated values of the conditional probability of detection for the three aerial observers extracted from the sub-sample of the 130 polygons monitored by aerial counting will then be extrapolated to all sampled sites to estimate the index of abundance over the whole of the Camargue and over the entire monitoring period. While this is the topic of a future study, this modelling approach already allowed estimating the corrected abundance index for the four protected areas considered. Our approach aimed to integrate two independent counting methods (ground and aerial censuses in our case) in order to estimate a conditional probability of detection which depends on the observers (for aerial censuses) and the protected areas (for ground censuses). We found that estimated abundance indices were consistently greater than bird numbers recorded during monitoring (i.e. counts were underestimated), which indicates that the model is able to estimate a conditional probability of detection to better understand population dynamics of wintering waterfowl. The indices of abundance extracted from our single species models are not comparable between species, because the inclusion of a species effect in a single model led to convergence problems that we were unable to solve, hence a model per species. However, the temporal dynamics between species can be compared.

One added value of our model is to combine count data from two protocols to estimate relative detection probability and abundance indices without physical capture of animals. Recent state-space models are usually based on a single count per time interval without accounting for detection probability, even conditional [25, 52]. Our model uses counts replicated in two dimensions (multiple sites and replicate observations per site), like *N*-mixture models [21], but with a more flexible framework. Assumptions of the *N*-mixture model require the implementation of rigorous monitoring protocols which are difficult to apply to most real-life wildlife monitoring programmes because of ecological, logistical or economic constraints [16, 26]. The application framework of our state-space model takes advantage of two independent counting protocols carried out by different monitoring programs to estimate a conditional detection probability. In doing so, our model estimates an abundance index, and not absolute abundance.

Sensitivity analyses did not reveal biased estimates in the differences between the conditional detection probabilities of the observers and protected areas, regardless of the value set for Réserve Naturelle des Marais du Vigueirat (see S3 Appendix). In this way, the model allowed us to correct the abundance index by shifts in the conditional detection probability and captured well the temporal dynamic observed in the count data. The abundance index estimated by the model shows that counts are often underestimated, albeit with an ecologically plausible credible interval. Previous research has shown that observers generally underestimate waterbird abundance during aerial surveys [53–56] and ground surveys [57], especially for large numbers. The next step will be to re-evaluate the trends of waterfowl at the scale of the Camargue from the sites’ estimated abundance indices by summing them and by modelling the temporal dynamics, although our model seemed too complex when it was tentatively applied to low abundance species, such as Northern Pintail (*Anas acuta*, on average 1 245 Northern Pintail ± 967 SD in January). Multi-species modelling techniques could be considered in order to use data from more abundant species to inform for rare species [58, 59].

Gregarious species are counted in groups, where it would be difficult to avoid double counts when measuring the size of flocks. The use of Student’s *t*-distribution to represent the observation error works for gregarious species with large flocks, considering false-positive errors (i.e. misidentification and double counts). A low number of degrees of freedom favours extreme values on the shape of the probability density function compared to a normal distribution, which allows for some possible large counting errors. Where counts are carried out on large flocks, individuals are not detected independently from each other. If they move in flocks, detection of one individual will make it more likely that other group members are also detected, which is a violation of the independence assumption of the observation process underlying the counts. The consequences of non-independent response variables are usually inflated sample size, underestimated variance, and overdispersion relative to the assumed model [60]. This is a recurrent problem, which has been dealt with by the choice of a beta-binomial distribution for the observation process of *N*-mixture models [61, 62]. This opens perspectives for future improvements of the current model towards this direction.

Counts of individuals in large groups lead to other limitations of the model, notably in the precise definition of the term *p* (Eq 7), which is considered here as a conditional detection probability. The difference in detection between observers may be due to their ability to detect birds (perception bias defined as the proportion of birds that are visible but not seen by observers, [63]) or to their ability to estimate groups of birds (group estimation error). Indeed, observers tend to underestimate the size of groups during counts, especially for large groups of individuals, as is the case during waterfowl counts in the Camargue [6]. In this context, it is difficult to clearly distinguish the influence of these two errors on the parameter *p* estimated by the model. For example, in the data analysed in this study, some sites have large open water areas where the error in estimating group size is likely to have a greater weight in the estimation of *p*, whereas the probability of detection is likely to be more important when counting sites with high vegetation cover, e.g. Tamarisk (*Tamarix gallica*), that can hide ducks. Since the probability of detection refers only to the perception bias, the availability of birds for both methods, if it varies between replicates, can lead to biases in the estimation of the model detection parameters. The availability of birds at a site can vary according to two elements: the number of days between the ground and aerial counts and the fact that some birds will be out of sight of the observers for the fixed-point ground method and therefore unavailable. We assume that these elements only slightly modify the number of birds available on the sites monitored in the Camargue. On average, there is a 3 days (± 3 days) difference between the counts of the two methods. It is unlikely that the number of individuals will vary greatly between replicates of counts as ducks are species that select day-roosts at the beginning of the wintering season when they arrive, and they remain faithful to them afterwards [45, 46, 64]. Furthermore, a preliminary analysis (correlation test) showed that the dependency between aerial and ground counts does not decrease the more lag days there are between the counts of the two methods. If a strong independence is observed between the data of the two counting methods when the number of lag days increases, we recommend considering the number of lag days in the linear predictor of the probability of detection. In addition, with respect to the potential effect of the sight limitations of the observers for ground counts, observers probably did not miss many birds because the sites were covered thoroughly during the counting surveys and there was good visibility over all ponds and marshes. For larger sites such as the Vaccarès pond in the Camargue National Nature Reserve, ground counts are carried out by boat to cover the entire pond and thus minimise bias due to differences in availability with aerial counts. As was done in this study, it is important to check these elements to ensure that the number of birds available does not vary too much between temporal replicates.

Our results show wide differences in conditional probability of detection between observers, protected areas and counting methods. Temporal and spatial heterogeneity in detection of an individual bird is subject to a variety of sources [11, 12, 14, 65]. In particular, our results show that the model estimates differences in detection rates between observers. These results add to the existing literature [15, 66], which shows that changes and differences in observer skills are an important potential source of bias as in the case of the BBS avian point count surveys [67]. Unfortunately, there was no overlap between the time periods covered by the different observers, hence no way to evaluate from their field data their individual detection error rates. However, if the differences between observers are only actual differences in numbers between periods of observer change (with observers having the same detection capabilities), similar fluctuations in numbers between periods would have been observed in the ground counts. The identities of ground observers were not recorded and hence included in the Camargue waterbird count databases, and too few monitoring programmes consider and record the identities of actual observers at national and international levels. It is important that changes in observers be integrated into most monitoring programmes in order to be incorporated into models able to estimate a probability of detection. Taking into account the differences between observers in the detection of individuals makes it possible to better identify variability sources and assess population trends and the environmental factors that impact them more accurately, in order to correctly design species-specific conservation actions [13, 25].

There were also large differences in detection between aerial and ground surveys. Ground surveys appeared to be closer to abundance indices than the aerial surveys were in the sense that counts were less underestimated compared to aerial survey. Actual count method is also an important known source of variation in bird detection rates [68], and numerous studies confirm that ground counts are less underestimated than aerial counts [56, 69–72]. Owing to their greater accuracy, many monitoring protocols actually use ground counts as a correction factor for aerial counts [73, 74]. For instance, breeding waterfowl population size estimates in North America rely on aerial surveys adjusted by visibility correction factors derived from counts made from the ground during which all waterfowl are assumed to have been detected [74]. However, ground counts are also subject to errors [57] and differences in the probability of detection between ground and aerial surveys vary according to several factors, although studies agree that generally more individuals are counted from the ground [72, 75, 76]. Terrestrial and aerial surveys both have their pros and cons, but are each associated with intrinsic limitations, in particular when it comes to detection probability of individuals and ability to estimate flock sizes, which vary among observers [6, 15, 66].

When the economic and logistical constraints do not allow for a protocol to estimate the probability of detection, the most reliable counting method is probably the one producing the most repeatably accurate index (i.e. where the proportion of individuals counted does not vary too much over time) in order to assess population trends. In the case of waterbird counts in the Camargue, our model failed in providing such information because it would have been necessary to compare the heterogeneity of the conditional probability of detection over time (from year to year) between methods. Globally, if the monitoring protocol is intended to meet local-scale objectives, for example within the framework of nature reserve management programmes, ground counts seem the most appropriate on the assumption that the probability of detection does not vary much over time. If the species are relatively easy to identify [56], aerial surveys are effective and cost-efficient for quantifying population size and habitat use of waterfowl and other waterbirds across vast (when the sample of areas that can be monitored from the ground are not representative of the study area) and especially inaccessible landscapes. Overall, the most appropriate choice of survey approach will be based on the need for precise and unbiased estimates, balanced with financial and logistical constraints. Also, the question of protocol homogenisation should be considered in the choice of the monitoring method if comparisons between sites are to be made, as in the case of the Pan-European Common Bird Monitoring Scheme (PECBMS), which produces national and supranational indexes [77].

Our results show that ground counts are more correlated than aerial counts with Mallard and Common Coot abundance indices, while the reverse is noted for Common Teal. In one case aerial counts provided more information to estimate the dynamics of Common Teal, whereas for Mallard and Common Coot ground counts provided more information. These results raise the interest of multiplying data sources in order to derive and improve a single reliable abundance indicator by providing more varied information. With the recent development of statistical analysis methods in ecology, the use of all available information collected by different monitoring protocols in combination with the most appropriate statistical approach is essential to improve the understanding of population dynamics in order to implement appropriate management actions.

## 5. Conclusion

We developed a state-space model to improve inference about spatial and temporal variation in relative wildlife abundance from heterogeneous sources of count data. Using statistical methods that take imperfect detection into account provides reliable trend estimates. Monitoring programs always face a trade-off between making the surveys feasible (economic and logistical constraints) and using statistical analyses that provide accurate estimates. Collecting data that allows accounting for imperfect detection by recent development of statistical analysis is not always straightforward and often difficult to apply in the field. The model-based data integration approach as described here seems to be a promising solution that takes advantage of as much as possible of the data collected from several methods that provide additional information. When the logistical constraints are too strong to set up a protocol that accounts for the detectability of individuals, the use of available data from several different protocols can still be integrated to reliability infer population dynamics and thus take appropriate conservation actions.

## Supporting information

S1 Appendix Specification of the prior distributions for the parameters in the models and Prior Predictive Check. (DOCX)

S2 Appendix JAGS code of the model. (DOCX)

S3 Appendix Sensitivity analysis. (DOCX)

S4 Appendix Bayesian fit analysis. (DOCX)
