# Supplementary material for: Accounting for detection probability with overestimation by integrating double monitoring programs over 40 years
Source: PLoS One. 2022 Mar 25;17(3):e0265730. doi: 10.1371/journal.pone.0265730 (PMC8956176; doi:10.1371/journal.pone.0265730)
Supplement: S1 Appendix — (DOCX) [file pone.0265730.s001.docx]

**Electronic supplementary material**

**Accounting for detection probability with overestimation by integrating double monitoring programs over 40 years**

David Vallecillo^1,2*^, Matthieu Guillemain^2^, Matthieu Authier^3^, Colin Bouchard^4^, Damien Cohez^1^, Emmanuel Vialet^5^, Grégoire Massez^6^, Philippe Vandewalle^7^, Jocelyn Champagnon^1^

^1^ Tour du Valat, Research institute for the conservation of Mediterranean wetlands, Le Sambuc, 13200 Arles, France

^2^ OFB, Unité Avifaune migratrice, La Tour du Valat, Le Sambuc, 13200 Arles, France

^3^ Observatoire Pelagis, UMS 3462 CNRS-LRUniv ADERA, 17 000 La Rochelle, France

^4^ UMR Ecobiop, e2S, Université de Pau et Pays de l’Adour, INRAE, 64310 Saint-Pée sur Nivelle, France

^5^

^6^

^7^ SNPN-RNN de Camargue, 13 200 Arles, France

* Corresponding author

E-mail : [vallecillo@tourduvalat.org](mailto:vallecillo@tourduvalat.org)

**S1 Appendix**

**Specification of the prior distributions for the parameters in the models**

| Model | Prior distribution |
| --- | --- |
| State process | *mu* $\sim\text{Normal(0, 25)}$ |
|  | Unscaled_sigma2_site$\sim\text{Gamma(}\text{1.0, 1.0}\text{)}$ |
|  | Gam_site$\sim\text{Gamma(}\text{1.0, 1.0}\text{)}$ |
|  | $\text{σ}_{\text{site }}\text{= 1.0 × }\sqrt{\text{ }\frac{\text{Unscaled\_sigma2\_site}}{\text{Gam\_site}}\text{ }}$ |
|  | Unscaled_sigma2_year$\sim\text{Gamma(}\text{1.0, 1.0}\text{)}$ |
|  | Gam_year$\sim\text{Gamma(}\text{1.0, 1.0}\text{)}$ |
|  | $\text{σ}_{\text{year}}\text{ = log(1.05) × }\sqrt{\text{ }\frac{\text{Unscaled\_sigma2\_year}}{\text{Gam\_year}}\text{ }}$ |
|  | Prop ~ Uniform(0.0, 1.0) |
|  | Unscaled_sigma2_month$\sim\text{Gamma(}\text{1.0, 1.0}\text{)}$ |
|  | Gam_month$\sim\text{Gamma(}\text{1.0, 1.0}\text{)}$ |
|  | $\sigma_{\text{month1}}\text{= log(5.0) * }\sqrt{\text{Prop × }\frac{\text{Unscaled\_sigma2\_month}}{\text{Gam\_month}}\text{ }}$ |
|  | $\sigma_{\text{month2}}\text{= log(5.0) * }\sqrt{\text{(1 - Prop)}\text{ }\text{× }\frac{\text{Unscaled\_sigma2\_month}}{\text{Gam\_month}}\text{ }}$ |
|  | Unscaled_sigma2_res*_i_*$\sim\text{Gamma(}\text{1.0, 1.0}\text{)}$ |
|  | Gam_month*_i_*$\sim\text{Gamma(}\text{1.0, 1.0}\text{)}$ |
|  | ${\sigma_{\text{res}}}_{i} \text{= log(1.2)}\text{ }\text{* }\sqrt{\text{ }\frac{{Unscaled\_sigma2\_res}_{i}}{{Gam\_monthi}_{i}}\text{ }}$ |
| Detection process | $\text{β}_{\text{1}} \sim\text{ }\text{Student(0.0, 2.25, 7)}$ |
|  | $\text{β}_{\text{2}}=0$ |
|  | $\text{γ}_{\text{1,s,t1}}\sim\text{Normal(0}\text{.0}\text{, }\text{0.}\text{65)}$ |
|  | $\text{γ}_{\text{1,s,t2}}\sim\text{Normal(0}\text{.0}\text{, }\text{0.}\text{65)}$ |
|  | $\text{γ}_{\text{2,s1,t}}\sim\text{Normal(0.0, 1.33)}$ |
|  | $\text{γ}_{\text{2,s2,t}}\sim\text{Normal(0.0, 1.33)}$ |
|  | $\text{γ}_{\text{2,s3,t1}}\sim\text{Normal(0.0, 1.33)}$ |
|  | $\text{γ}_{\text{2,s3,t2}}\sim\text{Normal(0.0, 1.33)}$ |

**Prior Predictive Check**

What is the Prior Predictive Check?

This is a verification step. It allows us to highlight the adequacy between our model, the priors that we impose and the data on which we are going to work.

How does it work?

- the first step is to create a function that allows us to simulate data according to the model that will be used on our real dataset.

- we define the priors we want to use in our analysis.

- we draw values for each priors (10 000 simulations) within their distribution laws.

- we generate data with our simulation model for each combination of priors values.

- we compare the simulated data with the real data set.

If the majority of the simulated data falls within the same range of variation as the real dataset, then the defined priors are suitable for working with our waterbird count data. Care should be taken to ensure that the defined priors are not too informative or too uninformative.

Function to simulate the data

data.fn **<-** **function(**n_site **=** 40, #number of sites monitored

n_year **=** 44, #number of years monitored

n_month **=** 7, #number of month monitored

n_method **=** 2, #number of monitoring method

mu **=** log**(**500**)**,

sigma_site **=** log**(**2.00**)** **/** 2,

sigma_year **=** log**(**1.05**)** **/** 2,

sigma_month **=** log**(**1.1**)** **/** 2,

sigma_alpha_year **=** log**(**1.05**)** **/** 2,

detection **=** list**(**method1 **=** c**(**0.30, 0.25, 0.50**)**,

method2 **=** c**(**0.80, 0.40, 0.45, 0.85, 0.90**)**

**)**,

logit **=** **FALSE**,

prop_missing_data **=** **NULL**,

seed **=** **NULL**

**)** **{**

### for reproducibility

**if(!**is.null**(**seed**)** **&&** is.numeric**(**seed**))** **{**

set.seed**(**seed**)**

**}**

### abundance data for sites

log_N **<-** rnorm**(**n_site, mu, sigma_site**)**

data_trend **<-** data.frame**(**Site **=** rep**(**paste**(**"site", 1**:**n_site, sep**=**"_"**)**, each **=** n_year**)**,

Year **=** rep**(**1**:**n_year, n_site**)**,

N **=** rep**(**exp**(**log_N**)**, each **=** n_year**)**

**)**

log_r **<-** array**(**0, dim **=** c**(**n_site, n_year**))**

**for** **(**s **in** 1**:**n_site**){**

log_r**[**s, **-**1**]** **<-** rnorm**(**n_year **-** 1, 0, sigma_year**)**

log_r**[**s, **]** **<-** cumsum**(**log_r**[**s, **])** # random walk of order 1

**}**; rm**(**s**)**

**for** **(**s **in** 1**:**n_site**){**

**for** **(**t **in** 2**:**n_year**){**

data_trend**$**N**[(**s**-**1**)***n_year**+**t**]** **<-** **(**data_trend**$**N**[(**s**-**1**)***n_year**+**t**-**1**]** ***** exp**(**log_r**[**s,t**]))**

**}**

**}**; rm**(**s, t**)**

# p6_tn <- ggplot(data = data_trend,aes(group=as.factor(Site),colour=Site)) +

# geom_line(aes(x = Year,y=N)) +

# geom_hline(yintercept = 1, color = "black", linetype = "dotted") +

# scale_y_log10(name = "N", breaks = 10^c(0:6), labels = c(1, 10, quote(10^2), quote(10^3), quote(10^4), quote(10^5), quote(10^6))) +

# coord_cartesian(ylim = c(0.9, 1e6))+

# facet_wrap(~Site) + guides(color = "none") + ggtitle("B.")

# p6_tn

### intermensual stochasticity, seasonnal pattern

delta **<-** **function(**n_month, sigma_month**)** **{**

delta **<-** rep**(**0, n_month**)**

delta**[-**1**]** **<-** sigma_month ***** rnorm**(**n_month **-** 1**)** # random walk of order 1

return**(**cumsum**(**delta**))**

**}**

# plot(exp(delta(n_month = n_month, sigma_month = sigma_month)), type = 'l', ylim = c(0.8, 1.2), las = 1)

# for(i in 1:20) {lines(exp(delta(n_month = n_month, sigma_month = sigma_month)))}

### interannual stochasticity,annual pattern

alpha **<-** **function(**delta, n_year, sigma_alpha_year**)** **{**

n_month **<-** length**(**delta**)**

n **<-** n_month ***** n_year

return**(**t**(**replicate**(**n_year, delta **+** rnorm**(**n_month**)** ***** sigma_alpha_year**)))**

**}**

### visualization of the seasonal pattern

seasonal_pattern **<-** delta**(**n_month **=** n_month, sigma_month **=** sigma_month**)**

# plot(seasonal_pattern, type = 'l', ylim = c(-0.1, 0.1), las = 1, bty = 'n')

### visualization of the annual pattern

annual_pattern **<-** alpha**(**delta **=** seasonal_pattern, n_year **=** n_year, sigma_alpha_year **=** sigma_alpha_year**)**

# for(i in 1:nrow(annual_pattern)) {

# lines(annual_pattern[i,], col = grey(0.8))

# }

# lines(apply(annual_pattern, 2, mean), col = "midnightblue")

### abundance according to annual pattern for each site and each year

abund_site **<-** **function(**annual_pattern, abundance**)** **{**

N **<-** array**(NA**, dim **=** dim**(**annual_pattern**))**

N**[**, 1**]** **<-** abundance

**for(**t **in** 1**:**nrow**(**annual_pattern**))** **{**

N**[**t, **]** **<-** N**[**t, 1**]** ***** exp**(**annual_pattern**[**t, **])**

**}**

return**(**N**)**

**}**

### True abundance with annual pattern, ecological process

true_abund **<-** sapply**(**1**:**n_site,

**function(**site**)** **{**

abund_site**(**annual_pattern **=** annual_pattern, abundance **=** subset**(**data_trend, Site **==** paste**(**"site", site, sep **=** "_"**))$**N**)**

**}**,

simplify **=** "array"

**)**

### parameter of the linear predictor on the logit scale

##betac parameter

# from probability

# sanity check

**if(**all**(**unlist**(**detection**)** **<=** 1**)** **&&** all**(**unlist**(**detection**)** **>=** 0**)** **&&** logit**)** **{**

writeLines**(**"\tPlease check that detection parameters are on the logit scale"**)**

**}**

**if(**any**(**unlist**(**detection**)** **<=** 0**)** **&&** **!**logit**)** **{**

stop**(**"\tPlease check that detection parameters are on the probability scale"**)**

**}**

**if(**logit **==** **FALSE)** **{**

#aircraft method 1

Obs_1 **<-** detection**$**method1**[**1**]**

Obs_2 **<-** detection**$**method1**[**2**]**

Obs_3 **<-** detection**$**method1**[**3**]**

betac1 **<-** log**(**Obs_1**/** **(**1 **-** Obs_1**))**

first_change **<-** log**(**Obs_2**/** **(**1**-**Obs_2**))** **-** betac1

second_change **<-** log**(**Obs_3**/** **(**1**-**Obs_3**))** **-** betac1

#ground method 2

Vigueirat **<-** detection**$**method2**[**5**]**

Palissade **<-** detection**$**method2**[**1**]**

RéserveNationaleCamargue **<-** detection**$**method2**[**2**]**

TourduValat **<-** detection**$**method2**[**3**]**

TourduValatNew **<-** detection**$**method2**[**4**]**

betac2 **<-** log**(**Vigueirat**/(**1**-**Vigueirat**))**

Pali **<-** log**(**Palissade**/(**1**-**Palissade**))** **-** betac2

RNN **<-** log**(**RéserveNationaleCamargue**/(**1**-** RéserveNationaleCamargue**))** **-** betac2

TdV **<-** log**(**TourduValat**/(**1**-**TourduValat**))** **-** betac2

TdVNew **<-** log**(**TourduValatNew**/(**1**-**TourduValatNew**))** **-** betac2

**}**

**else** **{**

#aircraft method 1

betac1 **<-** detection**$**method1**[**1**]**

first_change **<-** detection**$**method1**[**2**]**

second_change **<-** detection**$**method1**[**3**]**

#ground method 2

betac2 **<-** detection**$**method2**[**5**]**

Pali **<-** detection**$**method2**[**1**]**

RNN **<-** detection**$**method2**[**2**]**

TdV **<-** detection**$**method2**[**3**]**

TdVNew **<-** detection**$**method2**[**4**]**

**}**

betac **<-** matrix**(NA**, ncol**=**2**)**

betac**[**, 1**]** **<-** betac1

betac**[**, 2**]** **<-** betac2

## gamma parameter

#aircraft method

gamma**<-**array**(NA**, c**(**n_site,n_year,n_method**))**

**for(**s **in** 1**:**n_site**)** **{**

**for(**t **in** 1**:**27**)** **{** gamma**[**s, t, 1**]** **<-** 0.0 **}**

**for(**t **in** 28**:**37**)** **{** gamma**[**s, t, 1**]** **<-** first_change **}**

**for(**t **in** 38**:**n_year**)** **{** gamma**[**s, t, 1**]** **<-** second_change **}**

**}**; rm**(**s, t**)**

#ground method

**for(**s **in** 1**:**7**)** **{**

**for(**t **in** 1**:**n_year**)** **{** gamma**[**s, t, 2**]** **<-** Pali **}**

**}**

**for(**s **in** 8**:**28**)** **{**

**for(**t **in** 1**:**n_year**)** **{** gamma**[**s, t, 2**]** **<-** RNN **}**

**}**

**for(**s **in** 29**:**38**)** **{**

**for(**t **in** 1**:**31**)** **{** gamma**[**s, t, 2**]** **<-** TdV **}**

**for(**t **in** 32**:**n_year**)** **{** gamma**[**s, t, 2**]** **<-** TdVNew **}**

**}**

**for(**s **in** 39**:**40**)** **{**

**for(**t **in** 1**:**n_year**)** **{** gamma**[**s, t, 2**]** **<-** 0.0 **}**

**}**; rm**(**s, t**)**

###linear predictor for detection probability

p **<-** array**(NA**, c**(**n_method,n_site,n_year**))**

**for(**s **in** 1**:**n_site**)** **{**

**for(**t **in** 1**:**n_year**)** **{**

**for(**i **in** 1**:**n_method**)** **{**

p**[**i, s, t**]** **<-** plogis**(**betac**[**i**]** **+** gamma**[**s, t, i**])**

**}**

**}**

**}**; rm**(**s, t, i**)**

### verif

# plogis(betac[1]+gamma[, 27, 1])

# p[1,,27]

# plogis(betac[1]+gamma[, 28, 1])

# p[1,,28]

# plogis(betac[2]+gamma[29:38,31, 2])

# p[2,29:38,31]

# plogis(betac[2]+gamma[29:38,32, 2])

# p[2,29:38,32]

### count data

y **<-** array**(NA**, c**(**n_method, n_month, n_site, n_year**))**

**for(**i **in** 1**:**n_method**)** **{**

**for(**m **in** 1**:**n_month**)** **{**

**for(**s **in** 1**:**n_site**)** **{**

**for(**t **in** 1**:**n_year**)** **{**

### sanity check

**if(**round**(**true_abund**[**t, m, s**])** **>** 0**)** **{**

y**[**i, m, s, t**]** **<-** rbinom**(**n **=** 1, size **=** round**(**true_abund**[**t, m, s**])**, prob **=** p**[**i, s, t**])**

**}**

**else** **{** y**[**i, m, s, t**]** **<-** 0 **}**

**}**

**}**

**}**

**}**; rm**(**i, m, s, t**)**

**if(**is.null**(**prop_missing_data**))** **{**

dd **<-** list**(**y_obs **=** y,

p **=** p

**)**

**}**

**else{**

**if(**prop_missing_data **>** 1 **|** prop_missing_data **<** 0**)** **{**

stop**(**"\t Proportion of missing data must be between 0 and 1"**)**

**}**

**else** **{**

miss_1 **<-** prop_missing_data **/** 3

miss_2 **<-** 2 ***** prop_missing_data **/** 3

n_obs **<-** n_month ***** n_site ***** n_year

missed_obs1 **<-** array**(**rbinom**(**n_obs, size **=** 1, prob **=** miss_1**)**, dim **=** c**(**n_month, n_site, n_year**))**

missed_obs2 **<-** array**(**rbinom**(**n_obs, size **=** 1, prob **=** miss_2**)**, dim **=** c**(**n_month, n_site, n_year**))**

y_obs **<-** y

y_obs**[**1, , , **]** **<-** y_obs**[**1, , , **]** ***** ifelse**(**missed_obs1 **==** 0, 1, **NA)**

y_obs**[**2, , , **]** **<-** y_obs**[**2, , , **]** ***** ifelse**(**missed_obs2 **==** 0, 1, **NA)**

dd **<-** list**(**y_obs **=** y_obs,

y_true **=** y,

p **=** p

**)**

**}**

**}**

return**(**list**(**data **=** dd,

param **=** list**(**true_abund **=** true_abund,

seasonal_pattern **=** seasonal_pattern,

annual_pattern **=** annual_pattern,

r **=** exp**(**log_r**)**

**)**,

hyperparam **=** list**(**betac **=** betac,

gamma **=** gamma,

sigma_site **=** sigma_site,

sigma_year **=** sigma_year,

sigma_month **=** sigma_month,

sigma_alpha_year **=** sigma_alpha_year

**)**#,

#plot = p6_tn

**)**

**)**

**}**

#

# test <- data.fn()

# test <- data.fn(prop_missing_data = 0.5) #total proportion of missing data

# test <- data.fn(detection = list(method1 = c(-1.5, -0.5, 0.2), method2 = c(-2.5, 1.8)), logit = TRUE)

# test <- data.fn(detection = list(method1 = c(-1.5, -0.5, 0.2), method2 = c(-2.5, 1.8)))

#

# rm(list = ls()[-match(x = c("datajags", "detection", "mat", "out", "sarcelle", "Simulateddata", "temp",

# "nb", "nc", "nt", "ni", "Obsdata", "observateur", "log_COUNT", "params.jags",

# "WorkDir", "data.fn"), table = ls())])

Code for Prior Predictive Check

#load the packages

library**(**dplyr**)**

library**(**gridExtra**)**

library**(**ggplot2**)**

####Prior predictive check

rm**(**list **=** ls**())**

n_sim **<-** 1e4 #number of simulation

n_site **<-** 40 #number of sites monitored

n_year **<-** 44 #number of years monitored

n_month **<-** 7 #number of month monitored

n_method **<-** 2 #number of monitoring method

####draw values for each priors (10 000 simulations) within their distribution laws####

#sigma_site

prior_site **<-** log**(**2**)** **/** 2

unscaled_sigma2_site **<-** rgamma**(**n_sim, 1.0, 1.0**)**

gam_site **<-** rgamma**(**n_sim, 1.0, 1.0**)**

sigma_site **<-** prior_site ***** sqrt**(**unscaled_sigma2_site **/** gam_site**)**

#hist(sigma_site)

#mu

mu **<-** sn**::**rst**(**n_sim, xi **=** log**(**500**)**, omega **=** log**(**5**)** **/** 2, nu **=** 7**)**

#hist(exp(mu))

#summary(exp(mu))

#abline(v=exp(quantile(mu, probs = 0.025)), col="blue")

#abline(v=exp(quantile(mu, probs = 0.975)), col="blue")

#check taille sites first year

Log_N1 **<-** rnorm**(**n_sim, mu, sigma_site**)**

# hist(Log_N1, breaks = 100)

# abline(v = quantile(Log_N1, probs = 0.025), col="blue")

# abline(v = quantile(Log_N1, probs = 0.975), col="blue")

# hist(exp(Log_N1), breaks = 1000)

#sigma_year

prior_year **<-** log**(**1.05**)** **/** 2

unscaled_sigma2_year **<-** rgamma**(**n_sim, 1.0, 1.0**)**

gam_year **<-** rgamma**(**n_sim, 1.0, 1.0**)**

sigma_year **<-** prior_year ***** sqrt**(**unscaled_sigma2_year **/** gam_year**)**

# hist(sigma_year)

# summary(sigma_year)

#check sigma_year

log_r **<-** rnorm **(**n_sim, 0, sigma_year**)**

# hist(exp(log_r), breaks = 1000)

# summary(exp(log_r))

# abline(v = exp(quantile(log_r, probs = 0.025)), col="blue")

# abline(v = exp(quantile(log_r, probs = 0.975)), col="blue")

#sigma_month et sigma_alpha_year

#sigma_alpha_year correspond sigma_month[2]

#sigma_month correspond sigma_month[1]

prop **<-** runif**(**n_sim,0.0, 1.0**)**

hist**(**prop**)**

unscaled_sigma2_month **<-** rgamma**(**n_sim,1.0, 1.0**)**

gam_month **<-** rgamma**(**n_sim,1.0, 1.0**)**

prior_month **<-** log**(**1.055**)**

sigma_month **<-** prior_month ***** sqrt**(**prop ***** unscaled_sigma2_month **/** gam_month**)**

sigma_alpha_year **<-** prior_month ***** sqrt**((**1 **-** prop**)** ***** unscaled_sigma2_month **/** gam_month**)**

hist**(**sigma_month**)**

hist**(**sigma_alpha_year**)**

#tau_res

prior_res **=** log**(**1.2**)**

unscaled_sigma2_res **<-** array**(NA**, c**(**2, n_sim**))**

gam_res **<-** array**(NA**, c**(**2, n_sim**))**

sigma_res **<-** array**(NA**, c**(**2, n_sim**))**

**for(**i **in** 1**:**2**)** **{**

unscaled_sigma2_res**[**i,**]** **<-** rgamma**(**n_sim, 1.0, 1.0**)**

gam_res**[**i,**]** **<-** rgamma**(**n_sim, 1.0, 1.0**)**

sigma_res**[**i,**]** **<-** prior_res ***** sqrt**(**unscaled_sigma2_res**[**i,**]** **/** gam_res**[**i,**])**

**}**

hist**(**sigma_res**[**1,**])**

hist**(**sigma_res**[**2,**])**

#detection process

betac **<-** replicate**(**2, sn**::**rst**(**n_sim, xi **=** 0, omega **=** 1.5, nu **=** 7**))**

Obs_1 **<-** rep**(**0, n_sim**)**

Obs_2 **<-** rnorm**(**n_sim**)** ***** log**(**5**)** **/** 2

Obs_3 **<-** rnorm**(**n_sim**)** ***** log**(**5**)** **/** 2

Vigueirat **<-** rep**(**0, n_sim**)**

Palissade **<-** rnorm**(**n_sim**)** ***** log**(**10**)** **/** 2

RéserveNationaleCamargue **<-** rnorm**(**n_sim**)** ***** log**(**10**)** **/** 2

TourduValat **<-** rnorm**(**n_sim**)** ***** log**(**10**)** **/** 2

TourduValatNew **<-** rnorm**(**n_sim**)** ***** log**(**10**)** **/** 2

####generate data with our simulation model for each combination of priors values####

setwd**(**WorkDir **<-** "C:/Users/Utilisateur/Desktop/Probadetec/probdetec/Final/david/Verif mod謥"**)**

source**(**paste**(**getwd**()**, "FunctionSimData2Source.r", sep **=** "/"**))**

priorpredcheck **<-** array**(NA**, c**(**n_method,n_month,n_site,n_year,n_sim**))**

**for(**i **in** 1**:**n_sim**){**

priorpredcheck**[**,,,,i**]<-**data.fn**(**mu **=** mu**[**i**]**,

sigma_site **=** sigma_site**[**i**]**,

sigma_year **=** sigma_year**[**i**]**,

sigma_month **=** sigma_month**[**i**]**,

sigma_alpha_year **=** sigma_alpha_year**[**i**]**,

detection **=** list**(**method1 **=** c**(**betac**[**i, 1**]**, Obs_2**[**i**]**, Obs_3**[**i**])**,

method2 **=** c**(**Palissade**[**i**]**, RéserveNationaleCamargue**[**i**]**, TourduValat**[**i**]**, TourduValatNew**[**i**]**, betac**[**i, 2**])**

**)**,

logit **=** **TRUE**

**)$**data**$**y_obs

**}**

#warnings()

str**(**priorpredcheck**[**1, 1, 1, 1, **])** #check the number of simulations

# hist(log1p(priorpredcheck[1, 1, 1, 1, ]))

# abline(v = log(1e3), col = "red")

#

# hist(log1p(priorpredcheck[1, 1, 1, 44, ]))

# abline(v = log(1e3), col = "red")

#

# hist(log1p(priorpredcheck[1, 1, 2, 1, ]))

# abline(v = log(1e3), col = "red")

#

# hist(log1p(priorpredcheck[1, 1, 2, 44, ]))

# abline(v = log(1e3), col = "red")

#

# hist(log1p(priorpredcheck[1, 2, 1, 1, ]))

# abline(v = log(1e3), col = "red")

#

# hist(log1p(priorpredcheck[1, 2, 1, 44, ]))

# abline(v = log(1e3), col = "red")

#

# hist(log1p(priorpredcheck[2, 2, 1, 44, ]))

# abline(v = log(1e3), col = "red")

#

# hist(log1p(priorpredcheck[2, 1, 1, 1, ]))

# abline(v = log(1e3), col = "red")

#

# hist(log1p(priorpredcheck[2, 7, 40, 44, ]))

####compare the simulated data with the real data set####

#average matrix per year per site per method and per simulation

Nobs **<-** array**(NA**, c**(**n_method,n_site,n_year,n_sim**))**

**for** **(**m **in** 1**:**n_method**){**

**for** **(**s **in** 1**:**n_site**){**

**for(**j **in** 1**:**n_year**){**

**for(**i **in** 1**:**n_sim**){**

Nobs**[**m,s,j,i**]** **<-** mean**(**priorpredcheck**[**m,,s,j,i**])**

**}**

**}**

**}**

**}**

# table(is.na(Nobs))

# str(Nobs)

# Nobs[1,1,1,]

#create the data frame

Pattaya **<-** **NULL**

**for** **(**m **in** 1**:**n_method**){**

**for(**j **in** 1**:**n_year**){**

**for** **(**s **in** 1**:**n_site**){**

Pitaya **<-** data.frame **(**N**=**Nobs**[**m,s,j,**]**, Method **=** m, Site **=** s, Year **=** j**)**

Pattaya **<-** rbind**(**Pattaya, Pitaya**)**

**}**

**}**

**}**

#head(Pattaya)

#str(Pattaya)

Pattaya **<-** cbind**(**Pattaya, rep**(**1**:**n_sim, 3520**))**

colnames**(**Pattaya**)** **<-** c**(**"N", "Method", "Site", "Year", "Sim"**)**

head**(**Pattaya**)**

Pattaya_1 **<-** dplyr**::**filter**(**Pattaya, Method **==** 1**)**

Pattaya_2 **<-** dplyr**::**filter**(**Pattaya, Method **==** 2**)**

head**(**Pattaya_1**)**

head**(**Pattaya_2**)**

#export and format the real dataset for each species#

AerialGroundCounts**<-**read.csv2**(**"Sol Avion.csv"**)**

head**(**AerialGroundCounts**)**

summary**(**AerialGroundCounts**)**

library**(**truncnorm**)**

####Common Teal#

AerialGroundCountsSarcelle **<-** AerialGroundCounts**[**AerialGroundCounts**$**Species **==** "Anas_crecca", **]**

summary**(**AerialGroundCountsSarcelle**)**

#Cr顴ion index_month

AerialGroundCountsSarcelle **<-** as.data.frame**(**AerialGroundCountsSarcelle**)**

class**(**AerialGroundCountsSarcelle**)**

str**(**AerialGroundCountsSarcelle**)**

AerialGroundCountsSarcelle **<-** AerialGroundCountsSarcelle %>%

mutate**(**Month **<-** as.character**(**Month**)**,

mois **=** factor**(**Month, levels **=** c**(**"9", "10", "11", "12", "1", "2", "3"**))**,

mois **=** as.numeric**(**mois**)**,

annee **=** Years

**)** %>%

arrange**(**annee, mois, Site_group**)**

###Data

log_COUNT **<-** array**(NA**, dim **=** c**(**2,

length**(**unique**(**AerialGroundCountsSarcelle**$**Month**))**,

length**(**unique**(**AerialGroundCountsSarcelle**$**Site_name**))**,

length**(**unique**(**AerialGroundCountsSarcelle**$**annee**))**

**)**

**)**

dd_methode1 **<-** subset**(**AerialGroundCountsSarcelle, select**=-**Ground_Count**)**

**for(**m **in** 1**:**length**(**unique**(**AerialGroundCountsSarcelle**$**mois**)))** **{**

dd_mois **<-** subset**(**dd_methode1, mois **==** unique**(**AerialGroundCountsSarcelle**$**mois**)[**m**])**

**for(**s **in** 1**:**length**(**unique**(**AerialGroundCountsSarcelle**$**Site_name**)))** **{**

dd_site **<-** subset**(**dd_mois, Site_name **==** unique**(**AerialGroundCountsSarcelle**$**Site_name**)[**s**])**

**for(**t **in** 1**:**length**(**unique**(**AerialGroundCountsSarcelle**$**annee**)))** **{**

dd_annee **<-** subset**(**dd_site, annee **==** unique**(**AerialGroundCountsSarcelle**$**annee**)[**t**])**

log_COUNT**[**1, m, s, t**]** **<-** ifelse**(**nrow**(**dd_annee**)** **==** 0, **NA**, log1p**(**dd_annee**$**Aerial_Count**))**

**}**

**}**

**}**; rm**(**m, s, t, dd_mois, dd_site, dd_annee, dd_methode1**)**

dd_methode2 **<-** subset**(**AerialGroundCountsSarcelle, select**=-**Aerial_Count**)**

**for(**m **in** 1**:**length**(**unique**(**AerialGroundCountsSarcelle**$**mois**)))** **{**

dd_mois **<-** subset**(**dd_methode2, mois **==** unique**(**AerialGroundCountsSarcelle**$**mois**)[**m**])**

**for(**s **in** 1**:**length**(**unique**(**AerialGroundCountsSarcelle**$**Site_name**)))** **{**

dd_site **<-** subset**(**dd_mois, Site_name **==** unique**(**AerialGroundCountsSarcelle**$**Site_name**)[**s**])**

**for(**t **in** 1**:**length**(**unique**(**AerialGroundCountsSarcelle**$**annee**)))** **{**

dd_annee **<-** subset**(**dd_site, annee **==** unique**(**AerialGroundCountsSarcelle**$**annee**)[**t**])**

log_COUNT**[**2, m, s, t**]** **<-** ifelse**(**nrow**(**dd_annee**)** **==** 0, **NA**, log1p**(**dd_annee**$**Ground_Count**))**

**}**

**}**

**}**; rm**(**m, s, t, dd_mois, dd_site, dd_annee, dd_methode2**)**

#v鲩f

AerialGroundCountsSarcelle

dim**(**log_COUNT**)**

unique**(**AerialGroundCountsSarcelle**$**Site_name**)**

unique**(**AerialGroundCountsSarcelle**$**annee**)**

# par(mfrow=c(1,2))

# datasimul <- t(priorpredcheck[1,4,4,,])

# plot(x=seq(1:52),rep.int(0,52),ylim = c(0, 10000),type="l",col=grey(1),lwd=2,xlab="Ann饳",ylab="Y_obs_sim");

# for(i in 1:length(datasimul[,1])){

# lines(1:52,datasimul[i,],col=grey(0.9));

# }

#

# datareal <- exp(log_COUNT[1,4,4,])

# plot(x=seq(1:52),datareal,ylim = c(0, 500),type="l",col="black",lwd=2,xlab="Ann饳",ylab="Y_obs")

#real data

#average matrix per year per site per method and per simu

Nobsreal **<-** array**(NA**, c**(**2,40,44**))**

**for** **(**m **in** 1**:**2**){**

**for(**j **in** 1**:**44**){**

**for** **(**s **in** 1**:**40**){**

Nobsreal**[**m,s,j**]** **<-** mean**(**expm1**(**log_COUNT**[**m,,s,j**])**, na.rm **=** **TRUE)**

**}**

**}**

**}**

table**(**is.na**(**Nobsreal**))**#NA quantity

#create the data frame

Realduck **<-** **NULL**

**for** **(**m **in** 1**:**2**){**

**for(**j **in** 1**:**44**){**

**for** **(**s **in** 1**:**40**){**

Real **<-** data.frame **(**N**=**Nobsreal**[**m,s,j**]**, Method **=** m, Site **=** s, Year **=** j**)**

Realduck **<-** rbind**(**Realduck, Real**)**

**}**

**}**

**}**

Realduck_1 **<-** dplyr**::**filter**(**Realduck, Method **==** 1**)**

Realduck_2 **<-** dplyr**::**filter**(**Realduck, Method **==** 2**)**

Pattaya_1**$**Site **<-** as.factor**(**Pattaya_1**$**Site**)**

Pattaya_2**$**Site **<-** as.factor**(**Pattaya_2**$**Site**)**

Realduck_1**$**Site **<-** as.factor**(**Realduck_1**$**Site**)**

Realduck_2**$**Site **<-** as.factor**(**Realduck_2**$**Site**)**

####Mallard#

unique**(**AerialGroundCounts**$**Species**)**

AerialGroundCountsColvert **<-** AerialGroundCounts**[**AerialGroundCounts**$**Species **==** "Anas_platyrhynchos", **]**

summary**(**AerialGroundCountsColvert**)**

#Cr顴ion index_month

AerialGroundCountsColvert **<-** as.data.frame**(**AerialGroundCountsColvert**)**

class**(**AerialGroundCountsColvert**)**

str**(**AerialGroundCountsColvert**)**

AerialGroundCountsColvert **<-** AerialGroundCountsColvert %>%

mutate**(**Month **<-** as.character**(**Month**)**,

mois **=** factor**(**Month, levels **=** c**(**"9", "10", "11", "12", "1", "2", "3"**))**,

mois **=** as.numeric**(**mois**)**,

annee **=** Years

**)** %>%

arrange**(**annee, mois, Site_group**)**

###Data

log_COUNT **<-** array**(NA**, dim **=** c**(**2,

length**(**unique**(**AerialGroundCountsColvert**$**Month**))**,

length**(**unique**(**AerialGroundCountsColvert**$**Site_name**))**,

length**(**unique**(**AerialGroundCountsColvert**$**annee**))**

**)**

**)**

dd_methode1 **<-** subset**(**AerialGroundCountsColvert, select**=-**Ground_Count**)**

**for(**m **in** 1**:**length**(**unique**(**AerialGroundCountsColvert**$**mois**)))** **{**

dd_mois **<-** subset**(**dd_methode1, mois **==** unique**(**AerialGroundCountsColvert**$**mois**)[**m**])**

**for(**s **in** 1**:**length**(**unique**(**AerialGroundCountsColvert**$**Site_name**)))** **{**

dd_site **<-** subset**(**dd_mois, Site_name **==** unique**(**AerialGroundCountsColvert**$**Site_name**)[**s**])**

**for(**t **in** 1**:**length**(**unique**(**AerialGroundCountsColvert**$**annee**)))** **{**

dd_annee **<-** subset**(**dd_site, annee **==** unique**(**AerialGroundCountsColvert**$**annee**)[**t**])**

log_COUNT**[**1, m, s, t**]** **<-** ifelse**(**nrow**(**dd_annee**)** **==** 0, **NA**, log1p**(**dd_annee**$**Aerial_Count**))**

**}**

**}**

**}**; rm**(**m, s, t, dd_mois, dd_site, dd_annee, dd_methode1**)**

dd_methode2 **<-** subset**(**AerialGroundCountsColvert, select**=-**Aerial_Count**)**

**for(**m **in** 1**:**length**(**unique**(**AerialGroundCountsColvert**$**mois**)))** **{**

dd_mois **<-** subset**(**dd_methode2, mois **==** unique**(**AerialGroundCountsColvert**$**mois**)[**m**])**

**for(**s **in** 1**:**length**(**unique**(**AerialGroundCountsColvert**$**Site_name**)))** **{**

dd_site **<-** subset**(**dd_mois, Site_name **==** unique**(**AerialGroundCountsColvert**$**Site_name**)[**s**])**

**for(**t **in** 1**:**length**(**unique**(**AerialGroundCountsColvert**$**annee**)))** **{**

dd_annee **<-** subset**(**dd_site, annee **==** unique**(**AerialGroundCountsColvert**$**annee**)[**t**])**

log_COUNT**[**2, m, s, t**]** **<-** ifelse**(**nrow**(**dd_annee**)** **==** 0, **NA**, log1p**(**dd_annee**$**Ground_Count**))**

**}**

**}**

**}**; rm**(**m, s, t, dd_mois, dd_site, dd_annee, dd_methode2**)**

#v鲩f

AerialGroundCountsColvert

dim**(**log_COUNT**)**

unique**(**AerialGroundCountsColvert**$**Site_name**)**

unique**(**AerialGroundCountsColvert**$**annee**)**

# par(mfrow=c(1,2))

# datasimul <- t(priorpredcheck[1,4,4,,])

# plot(x=seq(1:52),rep.int(0,52),ylim = c(0, 10000),type="l",col=grey(1),lwd=2,xlab="Ann饳",ylab="Y_obs_sim");

# for(i in 1:length(datasimul[,1])){

# lines(1:52,datasimul[i,],col=grey(0.9));

# }

#

# datareal <- exp(log_COUNT[1,4,4,])

# plot(x=seq(1:52),datareal,ylim = c(0, 500),type="l",col="black",lwd=2,xlab="Ann饳",ylab="Y_obs")

#real data

#average matrix per year per site per method and per simu

Nobsreal **<-** array**(NA**, c**(**2,40,44**))**

**for** **(**m **in** 1**:**2**){**

**for(**j **in** 1**:**44**){**

**for** **(**s **in** 1**:**40**){**

Nobsreal**[**m,s,j**]** **<-** mean**(**expm1**(**log_COUNT**[**m,,s,j**])**, na.rm **=** **TRUE)**

**}**

**}**

**}**

table**(**is.na**(**Nobsreal**))**#NA quantity

#create the data frame

Realduck **<-** **NULL**

**for** **(**m **in** 1**:**2**){**

**for(**j **in** 1**:**44**){**

**for** **(**s **in** 1**:**40**){**

Real **<-** data.frame **(**N**=**Nobsreal**[**m,s,j**]**, Method **=** m, Site **=** s, Year **=** j**)**

Realduck **<-** rbind**(**Realduck, Real**)**

**}**

**}**

**}**

Realduck_col_1 **<-** dplyr**::**filter**(**Realduck, Method **==** 1**)**

Realduck_col_2 **<-** dplyr**::**filter**(**Realduck, Method **==** 2**)**

Realduck_col_1**$**Site **<-** as.factor**(**Realduck_col_1**$**Site**)**

Realduck_col_2**$**Site **<-** as.factor**(**Realduck_col_2**$**Site**)**

####Common Coot#

unique**(**AerialGroundCounts**$**Species**)**

AerialGroundCountsFoulque **<-** AerialGroundCounts**[**AerialGroundCounts**$**Species **==** "Fulica_atra", **]**

summary**(**AerialGroundCountsFoulque**)**

#Cr顴ion index_month

AerialGroundCountsFoulque **<-** as.data.frame**(**AerialGroundCountsFoulque**)**

class**(**AerialGroundCountsFoulque**)**

str**(**AerialGroundCountsFoulque**)**

AerialGroundCountsFoulque **<-** AerialGroundCountsFoulque %>%

mutate**(**Month **<-** as.character**(**Month**)**,

mois **=** factor**(**Month, levels **=** c**(**"9", "10", "11", "12", "1", "2", "3"**))**,

mois **=** as.numeric**(**mois**)**,

annee **=** Years

**)** %>%

arrange**(**annee, mois, Site_group**)**

###Data

log_COUNT **<-** array**(NA**, dim **=** c**(**2,

length**(**unique**(**AerialGroundCountsFoulque**$**Month**))**,

length**(**unique**(**AerialGroundCountsFoulque**$**Site_name**))**,

length**(**unique**(**AerialGroundCountsFoulque**$**annee**))**

**)**

**)**

dd_methode1 **<-** subset**(**AerialGroundCountsFoulque, select**=-**Ground_Count**)**

**for(**m **in** 1**:**length**(**unique**(**AerialGroundCountsFoulque**$**mois**)))** **{**

dd_mois **<-** subset**(**dd_methode1, mois **==** unique**(**AerialGroundCountsFoulque**$**mois**)[**m**])**

**for(**s **in** 1**:**length**(**unique**(**AerialGroundCountsFoulque**$**Site_name**)))** **{**

dd_site **<-** subset**(**dd_mois, Site_name **==** unique**(**AerialGroundCountsFoulque**$**Site_name**)[**s**])**

**for(**t **in** 1**:**length**(**unique**(**AerialGroundCountsFoulque**$**annee**)))** **{**

dd_annee **<-** subset**(**dd_site, annee **==** unique**(**AerialGroundCountsFoulque**$**annee**)[**t**])**

log_COUNT**[**1, m, s, t**]** **<-** ifelse**(**nrow**(**dd_annee**)** **==** 0, **NA**, log1p**(**dd_annee**$**Aerial_Count**))**

**}**

**}**

**}**; rm**(**m, s, t, dd_mois, dd_site, dd_annee, dd_methode1**)**

dd_methode2 **<-** subset**(**AerialGroundCountsFoulque, select**=-**Aerial_Count**)**

**for(**m **in** 1**:**length**(**unique**(**AerialGroundCountsFoulque**$**mois**)))** **{**

dd_mois **<-** subset**(**dd_methode2, mois **==** unique**(**AerialGroundCountsFoulque**$**mois**)[**m**])**

**for(**s **in** 1**:**length**(**unique**(**AerialGroundCountsFoulque**$**Site_name**)))** **{**

dd_site **<-** subset**(**dd_mois, Site_name **==** unique**(**AerialGroundCountsFoulque**$**Site_name**)[**s**])**

**for(**t **in** 1**:**length**(**unique**(**AerialGroundCountsFoulque**$**annee**)))** **{**

dd_annee **<-** subset**(**dd_site, annee **==** unique**(**AerialGroundCountsFoulque**$**annee**)[**t**])**

log_COUNT**[**2, m, s, t**]** **<-** ifelse**(**nrow**(**dd_annee**)** **==** 0, **NA**, log1p**(**dd_annee**$**Ground_Count**))**

**}**

**}**

**}**; rm**(**m, s, t, dd_mois, dd_site, dd_annee, dd_methode2**)**

#v鲩f

AerialGroundCountsFoulque

dim**(**log_COUNT**)**

unique**(**AerialGroundCountsFoulque**$**Site_name**)**

unique**(**AerialGroundCountsFoulque**$**annee**)**

# par(mfrow=c(1,2))

# datasimul <- t(priorpredcheck[1,4,4,,])

# plot(x=seq(1:52),rep.int(0,52),ylim = c(0, 10000),type="l",col=grey(1),lwd=2,xlab="Ann饳",ylab="Y_obs_sim");

# for(i in 1:length(datasimul[,1])){

# lines(1:52,datasimul[i,],col=grey(0.9));

# }

#

# datareal <- exp(log_COUNT[1,4,4,])

# plot(x=seq(1:52),datareal,ylim = c(0, 500),type="l",col="black",lwd=2,xlab="Ann饳",ylab="Y_obs")

#real data

#average matrix per year per site per method and per simu

Nobsreal **<-** array**(NA**, c**(**2,40,44**))**

**for** **(**m **in** 1**:**2**){**

**for(**j **in** 1**:**44**){**

**for** **(**s **in** 1**:**40**){**

Nobsreal**[**m,s,j**]** **<-** mean**(**expm1**(**log_COUNT**[**m,,s,j**])**, na.rm **=** **TRUE)**

**}**

**}**

**}**

table**(**is.na**(**Nobsreal**))**#NA quantity

#create the data frame

Realduck **<-** **NULL**

**for** **(**m **in** 1**:**2**){**

**for(**j **in** 1**:**44**){**

**for** **(**s **in** 1**:**40**){**

Real **<-** data.frame **(**N**=**Nobsreal**[**m,s,j**]**, Method **=** m, Site **=** s, Year **=** j**)**

Realduck **<-** rbind**(**Realduck, Real**)**

**}**

**}**

**}**

Realduck_fou_1 **<-** dplyr**::**filter**(**Realduck, Method **==** 1**)**

Realduck_fou_2 **<-** dplyr**::**filter**(**Realduck, Method **==** 2**)**

Realduck_fou_1**$**Site **<-** as.factor**(**Realduck_fou_1**$**Site**)**

Realduck_fou_2**$**Site **<-** as.factor**(**Realduck_fou_2**$**Site**)**

#graphic representation of the results

# P1 <- ggplot() +

# geom_line(data = Pattaya_1, aes(x = Year, y = N, group = Sim), alpha = 0.01) +

# geom_line(data = Realduck_1, aes(x = Year, y = N), color = "red") +

# facet_grid(~ Site) +

# coord_cartesian(xlim = c(1,44), ylim = c(0,10000)) +

# theme_bw()

#

# P2 <- ggplot() +

# geom_line(data = Pattaya_2, aes(x = Year, y = N, group = Sim), alpha = 0.01) +

# geom_line(data = Realduck_2, aes(x = Year, y = N), color = "red") +

# facet_grid(~ Site) +

# coord_cartesian(xlim = c(1,44), ylim = c(0,10000)) +

# theme_bw()

#

# library(gridExtra)

# grid.arrange(P1, P2, nrow = 2)

###decomposition of the graphic window on some sites

#sites 1 to 5

P1 **<-** ggplot**(**subset**(**Pattaya_1, Site **==** "1" **|**

Site **==** "2" **|**

Site **==** "3" **|**

Site **==** "4" **|**

Site **==** "5" **))** **+**

geom_line**(**aes**(**x **=** Year, y **=** N, group **=** Sim**)**, alpha **=** 0.01**)** **+**

geom_line**(**data **=**subset**(**Realduck_1,Site **==** "1" **|** Site **==** "2" **|** Site **==** "3" **|** Site **==** "4" **|** Site **==** "5" **)** , aes**(**x **=** Year, y **=** N**)**, color **=** "red"**)** **+**

geom_line**(**data **=**subset**(**Realduck_col_1,Site **==** "1" **|** Site **==** "2" **|** Site **==** "3" **|** Site **==** "4" **|** Site **==** "5" **)** , aes**(**x **=** Year, y **=** N**)**, color **=** "blue"**)** **+**

geom_line**(**data **=**subset**(**Realduck_fou_1,Site **==** "1" **|** Site **==** "2" **|** Site **==** "3" **|** Site **==** "4" **|** Site **==** "5" **)** , aes**(**x **=** Year, y **=** N**)**, color **=** "yellow"**)** **+**

facet_grid**(~** Site**)** **+**

coord_cartesian**(**xlim **=** c**(**1,44**)**, ylim **=** c**(**0,10000**))** **+**

theme_bw**()**

P1

P2 **<-** ggplot**(**subset**(**Pattaya_2, Site **==** "1" **|**

Site **==** "2" **|**

Site **==** "3" **|**

Site **==** "4" **|**

Site **==** "5" **))** **+**

geom_line**(**aes**(**x **=** Year, y **=** N, group **=** Sim**)**, alpha **=** 0.01**)** **+**

geom_line**(**data **=**subset**(**Realduck_2,Site **==** "1" **|** Site **==** "2" **|** Site **==** "3" **|** Site **==** "4" **|** Site **==** "5" **)** , aes**(**x **=** Year, y **=** N**)**, color **=** "red"**)** **+**

geom_line**(**data **=**subset**(**Realduck_col_2,Site **==** "1" **|** Site **==** "2" **|** Site **==** "3" **|** Site **==** "4" **|** Site **==** "5" **)** , aes**(**x **=** Year, y **=** N**)**, color **=** "blue"**)** **+**

geom_line**(**data **=**subset**(**Realduck_fou_2,Site **==** "1" **|** Site **==** "2" **|** Site **==** "3" **|** Site **==** "4" **|** Site **==** "5" **)** , aes**(**x **=** Year, y **=** N**)**, color **=** "yellow"**)** **+**

facet_grid**(~** Site**)** **+**

coord_cartesian**(**xlim **=** c**(**1,44**)**, ylim **=** c**(**0,10000**))** **+**

theme_bw**()**

P2

grid.arrange**(**P1, P2, nrow **=** 2**)**

#sites 6 to 10

P1 **<-** ggplot**(**subset**(**Pattaya_1, Site **==** "6" **|**

Site **==** "7" **|**

Site **==** "8" **|**

Site **==** "9" **|**

Site **==** "10" **))** **+**

geom_line**(**aes**(**x **=** Year, y **=** N, group **=** Sim**)**, alpha **=** 0.01**)** **+**

geom_line**(**data **=**subset**(**Realduck_1,Site **==** "6" **|** Site **==** "7" **|** Site **==** "8" **|** Site **==** "9" **|** Site **==** "10" **)** , aes**(**x **=** Year, y **=** N**)**, color **=** "red"**)** **+**

geom_line**(**data **=**subset**(**Realduck_col_1,Site **==** "6" **|** Site **==** "7" **|** Site **==** "8" **|** Site **==** "9" **|** Site **==** "10" **)** , aes**(**x **=** Year, y **=** N**)**, color **=** "blue"**)** **+**

geom_line**(**data **=**subset**(**Realduck_fou_1,Site **==** "6" **|** Site **==** "7" **|** Site **==** "8" **|** Site **==** "9" **|** Site **==** "10" **)** , aes**(**x **=** Year, y **=** N**)**, color **=** "yellow"**)** **+**

facet_grid**(~** Site**)** **+**

coord_cartesian**(**xlim **=** c**(**1,44**)**, ylim **=** c**(**0,10000**))** **+**

theme_bw**()**

P2 **<-** ggplot**(**subset**(**Pattaya_2, Site **==** "6" **|**

Site **==** "7" **|**

Site **==** "8" **|**

Site **==** "9" **|**

Site **==** "5" **))** **+**

geom_line**(**aes**(**x **=** Year, y **=** N, group **=** Sim**)**, alpha **=** 0.01**)** **+**

geom_line**(**data **=**subset**(**Realduck_2,Site **==** "6" **|** Site **==** "7" **|** Site **==** "8" **|** Site **==** "9" **|** Site **==** "10" **)** , aes**(**x **=** Year, y **=** N**)**, color **=** "red"**)** **+**

geom_line**(**data **=**subset**(**Realduck_col_2,Site **==** "6" **|** Site **==** "7" **|** Site **==** "8" **|** Site **==** "9" **|** Site **==** "10" **)** , aes**(**x **=** Year, y **=** N**)**, color **=** "blue"**)** **+**

geom_line**(**data **=**subset**(**Realduck_fou_2,Site **==** "6" **|** Site **==** "7" **|** Site **==** "8" **|** Site **==** "9" **|** Site **==** "10" **)** , aes**(**x **=** Year, y **=** N**)**, color **=** "yellow"**)** **+**

facet_grid**(~** Site**)** **+**

coord_cartesian**(**xlim **=** c**(**1,44**)**, ylim **=** c**(**0,10000**))** **+**

theme_bw**()**

grid.arrange**(**P1, P2, nrow **=** 2**)**

#sites 11 to 15

P1 **<-** ggplot**(**subset**(**Pattaya_1, Site **==** "11" **|**

Site **==** "12" **|**

Site **==** "13" **|**

Site **==** "14" **|**

Site **==** "15" **))** **+**

geom_line**(**aes**(**x **=** Year, y **=** N, group **=** Sim**)**, alpha **=** 0.01**)** **+**

geom_line**(**data **=**subset**(**Realduck_1,Site **==** "11" **|** Site **==** "12" **|** Site **==** "13" **|** Site **==** "14" **|** Site **==** "15" **)** , aes**(**x **=** Year, y **=** N**)**, color **=** "red"**)** **+**

geom_line**(**data **=**subset**(**Realduck_col_1,Site **==** "11" **|** Site **==** "12" **|** Site **==** "13" **|** Site **==** "14" **|** Site **==** "15" **)** , aes**(**x **=** Year, y **=** N**)**, color **=** "blue"**)** **+**

geom_line**(**data **=**subset**(**Realduck_fou_1,Site **==** "11" **|** Site **==** "12" **|** Site **==** "13" **|** Site **==** "14" **|** Site **==** "15" **)** , aes**(**x **=** Year, y **=** N**)**, color **=** "yellow"**)** **+**

facet_grid**(~** Site**)** **+**

coord_cartesian**(**xlim **=** c**(**1,44**)**, ylim **=** c**(**0,10000**))** **+**

theme_bw**()**

P2 **<-** ggplot**(**subset**(**Pattaya_2, Site **==** "11" **|**

Site **==** "12" **|**

Site **==** "13" **|**

Site **==** "14" **|**

Site **==** "15" **))** **+**

geom_line**(**aes**(**x **=** Year, y **=** N, group **=** Sim**)**, alpha **=** 0.01**)** **+**

geom_line**(**data **=**subset**(**Realduck_2,Site **==** "11" **|** Site **==** "12" **|** Site **==** "13" **|** Site **==** "14" **|** Site **==** "15" **)** , aes**(**x **=** Year, y **=** N**)**, color **=** "red"**)** **+**

geom_line**(**data **=**subset**(**Realduck_col_2,Site **==** "11" **|** Site **==** "12" **|** Site **==** "13" **|** Site **==** "14" **|** Site **==** "15" **)** , aes**(**x **=** Year, y **=** N**)**, color **=** "blue"**)** **+**

geom_line**(**data **=**subset**(**Realduck_fou_2,Site **==** "11" **|** Site **==** "12" **|** Site **==** "13" **|** Site **==** "14" **|** Site **==** "15" **)** , aes**(**x **=** Year, y **=** N**)**, color **=** "yellow"**)** **+**

facet_grid**(~** Site**)** **+**

coord_cartesian**(**xlim **=** c**(**1,44**)**, ylim **=** c**(**0,10000**))** **+**

theme_bw**()**

grid.arrange**(**P1, P2, nrow **=** 2**)**

#sites 16 yo 20

P1 **<-** ggplot**(**subset**(**Pattaya_1, Site **==** "16" **|**

Site **==** "17" **|**

Site **==** "18" **|**

Site **==** "19" **|**

Site **==** "20" **))** **+**

geom_line**(**aes**(**x **=** Year, y **=** N, group **=** Sim**)**, alpha **=** 0.01**)** **+**

geom_line**(**data **=**subset**(**Realduck_1,Site **==** "16" **|** Site **==** "17" **|** Site **==** "18" **|** Site **==** "19" **|** Site **==** "20" **)** , aes**(**x **=** Year, y **=** N**)**, color **=** "red"**)** **+**

geom_line**(**data **=**subset**(**Realduck_col_1,Site **==** "16" **|** Site **==** "17" **|** Site **==** "18" **|** Site **==** "19" **|** Site **==** "20" **)** , aes**(**x **=** Year, y **=** N**)**, color **=** "blue"**)** **+**

geom_line**(**data **=**subset**(**Realduck_fou_1,Site **==** "16" **|** Site **==** "17" **|** Site **==** "18" **|** Site **==** "19" **|** Site **==** "20" **)** , aes**(**x **=** Year, y **=** N**)**, color **=** "yellow"**)** **+**

facet_grid**(~** Site**)** **+**

coord_cartesian**(**xlim **=** c**(**1,44**)**, ylim **=** c**(**0,10000**))** **+**

theme_bw**()**

P2 **<-** ggplot**(**subset**(**Pattaya_2, Site **==** "16" **|**

Site **==** "17" **|**

Site **==** "18" **|**

Site **==** "19" **|**

Site **==** "20" **))** **+**

geom_line**(**aes**(**x **=** Year, y **=** N, group **=** Sim**)**, alpha **=** 0.01**)** **+**

geom_line**(**data **=**subset**(**Realduck_2,Site **==** "16" **|** Site **==** "17" **|** Site **==** "18" **|** Site **==** "19" **|** Site **==** "20" **)** , aes**(**x **=** Year, y **=** N**)**, color **=** "red"**)** **+**

geom_line**(**data **=**subset**(**Realduck_col_2,Site **==** "16" **|** Site **==** "17" **|** Site **==** "18" **|** Site **==** "19" **|** Site **==** "20" **)** , aes**(**x **=** Year, y **=** N**)**, color **=** "blue"**)** **+**

geom_line**(**data **=**subset**(**Realduck_fou_2,Site **==** "16" **|** Site **==** "17" **|** Site **==** "18" **|** Site **==** "19" **|** Site **==** "20" **)** , aes**(**x **=** Year, y **=** N**)**, color **=** "yellow"**)** **+**

facet_grid**(~** Site**)** **+**

coord_cartesian**(**xlim **=** c**(**1,44**)**, ylim **=** c**(**0,10000**))** **+**

theme_bw**()**

grid.arrange**(**P1, P2, nrow **=** 2**)**

#sites 21 to 25

P1 **<-** ggplot**(**subset**(**Pattaya_1, Site **==** "21" **|**

Site **==** "22" **|**

Site **==** "23" **|**

Site **==** "24" **|**

Site **==** "25" **))** **+**

geom_line**(**aes**(**x **=** Year, y **=** N, group **=** Sim**)**, alpha **=** 0.01**)** **+**

geom_line**(**data **=**subset**(**Realduck_1,Site **==** "21" **|** Site **==** "22" **|** Site **==** "23" **|** Site **==** "24" **|** Site **==** "25" **)** , aes**(**x **=** Year, y **=** N**)**, color **=** "red"**)** **+**

geom_line**(**data **=**subset**(**Realduck_col_1,Site **==** "21" **|** Site **==** "22" **|** Site **==** "23" **|** Site **==** "24" **|** Site **==** "25" **)** , aes**(**x **=** Year, y **=** N**)**, color **=** "blue"**)** **+**

geom_line**(**data **=**subset**(**Realduck_fou_1,Site **==** "21" **|** Site **==** "22" **|** Site **==** "23" **|** Site **==** "24" **|** Site **==** "25" **)** , aes**(**x **=** Year, y **=** N**)**, color **=** "yellow"**)** **+**

facet_grid**(~** Site**)** **+**

coord_cartesian**(**xlim **=** c**(**1,44**)**, ylim **=** c**(**0,10000**))** **+**

theme_bw**()**

P2 **<-** ggplot**(**subset**(**Pattaya_2, Site **==** "21" **|**

Site **==** "22" **|**

Site **==** "23" **|**

Site **==** "24" **|**

Site **==** "25" **))** **+**

geom_line**(**aes**(**x **=** Year, y **=** N, group **=** Sim**)**, alpha **=** 0.01**)** **+**

geom_line**(**data **=**subset**(**Realduck_2,Site **==** "21" **|** Site **==** "22" **|** Site **==** "23" **|** Site **==** "24" **|** Site **==** "25" **)** , aes**(**x **=** Year, y **=** N**)**, color **=** "red"**)** **+**

geom_line**(**data **=**subset**(**Realduck_col_2,Site **==** "21" **|** Site **==** "22" **|** Site **==** "23" **|** Site **==** "24" **|** Site **==** "25" **)** , aes**(**x **=** Year, y **=** N**)**, color **=** "blue"**)** **+**

geom_line**(**data **=**subset**(**Realduck_fou_2,Site **==** "21" **|** Site **==** "22" **|** Site **==** "23" **|** Site **==** "24" **|** Site **==** "25" **)** , aes**(**x **=** Year, y **=** N**)**, color **=** "yellow"**)** **+**

facet_grid**(~** Site**)** **+**

coord_cartesian**(**xlim **=** c**(**1,44**)**, ylim **=** c**(**0,10000**))** **+**

theme_bw**()**

grid.arrange**(**P1, P2, nrow **=** 2**)**

#sites 26 to 30

P1 **<-** ggplot**(**subset**(**Pattaya_1, Site **==** "26" **|**

Site **==** "27" **|**

Site **==** "28" **|**

Site **==** "29" **|**

Site **==** "30" **))** **+**

geom_line**(**aes**(**x **=** Year, y **=** N, group **=** Sim**)**, alpha **=** 0.01**)** **+**

geom_line**(**data **=**subset**(**Realduck_1,Site **==** "26" **|** Site **==** "27" **|** Site **==** "28" **|** Site **==** "29" **|** Site **==** "30" **)** , aes**(**x **=** Year, y **=** N**)**, color **=** "red"**)** **+**

geom_line**(**data **=**subset**(**Realduck_col_1,Site **==** "26" **|** Site **==** "27" **|** Site **==** "28" **|** Site **==** "29" **|** Site **==** "30" **)** , aes**(**x **=** Year, y **=** N**)**, color **=** "blue"**)** **+**

geom_line**(**data **=**subset**(**Realduck_fou_1,Site **==** "26" **|** Site **==** "27" **|** Site **==** "28" **|** Site **==** "29" **|** Site **==** "30" **)** , aes**(**x **=** Year, y **=** N**)**, color **=** "yellow"**)** **+**

facet_grid**(~** Site**)** **+**

coord_cartesian**(**xlim **=** c**(**1,44**)**, ylim **=** c**(**0,10000**))** **+**

theme_bw**()**

P2 **<-** ggplot**(**subset**(**Pattaya_2, Site **==** "26" **|**

Site **==** "27" **|**

Site **==** "28" **|**

Site **==** "29" **|**

Site **==** "30" **))** **+**

geom_line**(**aes**(**x **=** Year, y **=** N, group **=** Sim**)**, alpha **=** 0.01**)** **+**

geom_line**(**data **=**subset**(**Realduck_2,Site **==** "26" **|** Site **==** "27" **|** Site **==** "28" **|** Site **==** "29" **|** Site **==** "30" **)** , aes**(**x **=** Year, y **=** N**)**, color **=** "red"**)** **+**

geom_line**(**data **=**subset**(**Realduck_col_2,Site **==** "26" **|** Site **==** "27" **|** Site **==** "28" **|** Site **==** "29" **|** Site **==** "30" **)** , aes**(**x **=** Year, y **=** N**)**, color **=** "blue"**)** **+**

geom_line**(**data **=**subset**(**Realduck_fou_2,Site **==** "26" **|** Site **==** "27" **|** Site **==** "28" **|** Site **==** "29" **|** Site **==** "30" **)** , aes**(**x **=** Year, y **=** N**)**, color **=** "yellow"**)** **+**

facet_grid**(~** Site**)** **+**

coord_cartesian**(**xlim **=** c**(**1,44**)**, ylim **=** c**(**0,10000**))** **+**

theme_bw**()**

grid.arrange**(**P1, P2, nrow **=** 2**)**

#sites 31 to 35

P1 **<-** ggplot**(**subset**(**Pattaya_1, Site **==** "31" **|**

Site **==** "32" **|**

Site **==** "33" **|**

Site **==** "34" **|**

Site **==** "35" **))** **+**

geom_line**(**aes**(**x **=** Year, y **=** N, group **=** Sim**)**, alpha **=** 0.01**)** **+**

geom_line**(**data **=**subset**(**Realduck_1,Site **==** "31" **|** Site **==** "32" **|** Site **==** "33" **|** Site **==** "34" **|** Site **==** "35" **)** , aes**(**x **=** Year, y **=** N**)**, color **=** "red"**)** **+**

geom_line**(**data **=**subset**(**Realduck_col_1,Site **==** "31" **|** Site **==** "32" **|** Site **==** "33" **|** Site **==** "34" **|** Site **==** "35" **)** , aes**(**x **=** Year, y **=** N**)**, color **=** "blue"**)** **+**

geom_line**(**data **=**subset**(**Realduck_fou_1,Site **==** "31" **|** Site **==** "32" **|** Site **==** "33" **|** Site **==** "34" **|** Site **==** "35" **)** , aes**(**x **=** Year, y **=** N**)**, color **=** "yellow"**)** **+**

facet_grid**(~** Site**)** **+**

coord_cartesian**(**xlim **=** c**(**1,44**)**, ylim **=** c**(**0,10000**))** **+**

theme_bw**()**

P2 **<-** ggplot**(**subset**(**Pattaya_2, Site **==** "31" **|**

Site **==** "32" **|**

Site **==** "33" **|**

Site **==** "34" **|**

Site **==** "35" **))** **+**

geom_line**(**aes**(**x **=** Year, y **=** N, group **=** Sim**)**, alpha **=** 0.01**)** **+**

geom_line**(**data **=**subset**(**Realduck_2,Site **==** "31" **|** Site **==** "32" **|** Site **==** "33" **|** Site **==** "34" **|** Site **==** "35" **)** , aes**(**x **=** Year, y **=** N**)**, color **=** "red"**)** **+**

geom_line**(**data **=**subset**(**Realduck_col_2,Site **==** "31" **|** Site **==** "32" **|** Site **==** "33" **|** Site **==** "34" **|** Site **==** "35" **)** , aes**(**x **=** Year, y **=** N**)**, color **=** "blue"**)** **+**

geom_line**(**data **=**subset**(**Realduck_fou_2,Site **==** "31" **|** Site **==** "32" **|** Site **==** "33" **|** Site **==** "34" **|** Site **==** "35" **)** , aes**(**x **=** Year, y **=** N**)**, color **=** "yellow"**)** **+**

facet_grid**(~** Site**)** **+**

coord_cartesian**(**xlim **=** c**(**1,44**)**, ylim **=** c**(**0,10000**))** **+**

theme_bw**()**

grid.arrange**(**P1, P2, nrow **=** 2**)**

#sites 36 to 40

P1 **<-** ggplot**(**subset**(**Pattaya_1, Site **==** "36" **|**

Site **==** "37" **|**

Site **==** "38" **|**

Site **==** "39" **|**

Site **==** "40" **))** **+**

geom_line**(**aes**(**x **=** Year, y **=** N, group **=** Sim**)**, alpha **=** 0.01**)** **+**

geom_line**(**data **=**subset**(**Realduck_1,Site **==** "36" **|** Site **==** "37" **|** Site **==** "38" **|** Site **==** "39" **|** Site **==** "40" **)** , aes**(**x **=** Year, y **=** N**)**, color **=** "red"**)** **+**

geom_line**(**data **=**subset**(**Realduck_col_1,Site **==** "36" **|** Site **==** "37" **|** Site **==** "38" **|** Site **==** "39" **|** Site **==** "40" **)** , aes**(**x **=** Year, y **=** N**)**, color **=** "blue"**)** **+**

geom_line**(**data **=**subset**(**Realduck_fou_1,Site **==** "36" **|** Site **==** "37" **|** Site **==** "38" **|** Site **==** "39" **|** Site **==** "40" **)** , aes**(**x **=** Year, y **=** N**)**, color **=** "yellow"**)** **+**

facet_grid**(~** Site**)** **+**

coord_cartesian**(**xlim **=** c**(**1,44**)**, ylim **=** c**(**0,10000**))** **+**

theme_bw**()**

P2 **<-** ggplot**(**subset**(**Pattaya_2, Site **==** "36" **|**

Site **==** "37" **|**

Site **==** "38" **|**

Site **==** "39" **|**

Site **==** "40" **))** **+**

geom_line**(**aes**(**x **=** Year, y **=** N, group **=** Sim**)**, alpha **=** 0.01**)** **+**

geom_line**(**data **=**subset**(**Realduck_2,Site **==** "36" **|** Site **==** "37" **|** Site **==** "38" **|** Site **==** "39" **|** Site **==** "40" **)** , aes**(**x **=** Year, y **=** N**)**, color **=** "red"**)** **+**

geom_line**(**data **=**subset**(**Realduck_col_2,Site **==** "36" **|** Site **==** "37" **|** Site **==** "38" **|** Site **==** "39" **|** Site **==** "40" **)** , aes**(**x **=** Year, y **=** N**)**, color **=** "blue"**)** **+**

geom_line**(**data **=**subset**(**Realduck_fou_2,Site **==** "36" **|** Site **==** "37" **|** Site **==** "38" **|** Site **==** "39" **|** Site **==** "40" **)** , aes**(**x **=** Year, y **=** N**)**, color **=** "yellow"**)** **+**

facet_grid**(~** Site**)** **+**

coord_cartesian**(**xlim **=** c**(**1,44**)**, ylim **=** c**(**0,10000**))** **+**

theme_bw**()**

grid.arrange**(**P1, P2, nrow **=** 2**)**
