# Supplementary material for: Accounting for detection probability with overestimation by integrating double monitoring programs over 40 years
Source: PLoS One. 2022 Mar 25;17(3):e0265730. doi: 10.1371/journal.pone.0265730 (PMC8956176; doi:10.1371/journal.pone.0265730)
Supplement: S2 Appendix — (DOCX) [file pone.0265730.s002.docx]

**Electronic supplementary material**

**Accounting for detection probability with overestimation by integrating double monitoring programs over 40 years**

David Vallecillo^1,2*^, Matthieu Guillemain^2^, Matthieu Authier^3^, Colin Bouchard^4^, Damien Cohez^1^, Emmanuel Vialet^5^, Grégoire Massez^6^, Philippe Vandewalle^7^, Jocelyn Champagnon^1^

^1^ Tour du Valat, Research institute for the conservation of Mediterranean wetlands, Le Sambuc, 13200 Arles, France

^2^ OFB, Unité Avifaune migratrice, La Tour du Valat, Le Sambuc, 13200 Arles, France

^3^ Observatoire Pelagis, UMS 3462 CNRS-LRUniv ADERA, 17 000 La Rochelle, France

^4^ UMR Ecobiop, e2S, Université de Pau et Pays de l’Adour, INRAE, 64310 Saint-Pée sur Nivelle, France

^5^

^6^

^7^ SNPN-RNN de Camargue, 13 200 Arles, France

* Corresponding author

E-mail : [vallecillo@tourduvalat.org](mailto:vallecillo@tourduvalat.org)

**S2 Appendix**

**JAGS code of the model**

# D. Vallecillo, M. Authier, C. Bouchard, M. Guillemain & J. Champagnon, Mars 2021

# notation used

# DATA

# n_site : number of sites

# n_month : number of months

# n_year : number of year

# log_COUNT is an array of dim [n_month, n_site, n_year]

model {

#### LIKELIHOOD ####

for(s in 1:n_site) {

### Initial Condition

logN[s] ~ dnorm(mu, tau_site)

N[s, 1] <- exp(logN[s])

r[s, 1] <- 0.0 # for identifiability

### Rest of the process

for(t in 2:n_year) {

r[s, t] ~ dnorm(r[s, t-1], tau_year)

N[s, t] <- N[s, t-1] * exp(r[s, t])

for(m in 1:n_month) {

for(i in 1:2) {

log_COUNT[i, m, s, t] ~ dt(log(N[s, t]) + alpha[m, t] + log(p[i, s, t]), tau_res[i], 4)

}

}

}

}

delta[1] <- 0.0 # for identifiability

for(m in 2:n_month) {

delta[m] ~ dnorm(delta[m-1], tau_month[1])

}

for(t in 1:n_year) {

for(m in 1:n_month) {

alpha[m, t] ~ dnorm(delta[m], tau_month[2])

}

}

### Detection

for(s in 1:n_site) {

for(t in 1:n_year) {

for(m in 1:n_month) {

for(i in 1:2) {

p[i, s, t] <- ilogit(beta[i] + gamma[i, s, t])

}

}

}

}

#### PRIOR ####

mu ~ dnorm(0.0, 0.04) # a prior between 0 and 18000

tau_site <- pow(sigma_site, -2)

sigma_site <- prior_site * sqrt(unscaled_sigma2_site / gam_site)

unscaled_sigma2_site ~ dgamma(1.0, 1.0)

gam_site ~ dgamma(1.0, 1.0)

tau_year <- pow(sigma_year, -2)

sigma_year = prior_year * sqrt(unscaled_sigma2_year / gam_year)

unscaled_sigma2_year ~ dgamma(1.0, 1.0)

gam_year ~ dgamma(1.0, 1.0)

prop ~ dunif(0.0, 1.0)

tau_month[1] <- pow(sigma_month[1], -2)

tau_month[2] <- pow(sigma_month[2], -2)

sigma_month[1] <- prior_month * sqrt(prop * unscaled_sigma2_month / gam_month)

sigma_month[2] <- prior_month * sqrt((1 - prop) * unscaled_sigma2_month / gam_month)

unscaled_sigma2_month ~ dgamma(1.0, 1.0)

gam_month ~ dgamma(1.0, 1.0)

for(i in 1:2) {

tau_res[i] <- pow(sigma_res[i], -2)

sigma_res[i] <- prior_res * sqrt(unscaled_sigma2_res[i] / gam_res[i])

unscaled_sigma2_res[i] ~ dgamma(1.0, 1.0)

gam_res[i] ~ dgamma(1.0, 1.0)

}

### detection process

beta[1] <- beta_aerial # Aerial

beta_aerial ~ dt(0.0, 0.4444444, 7) # Aerial

beta[2] <- 0 # Ground

Réserve_Naturelle_des_Marais_du_Vigueirat <- 0.0

Marais_de_la_Palissade ~ dnorm(0.0, 0.75) # a priori new method can increase/decrease detection proba by a factor between 1/10 and 10

Réserve_Naturelle_Nationale_de_Camargue ~ dnorm(0.0, 0.75) # a priori new method can increase/decrease detection proba by a factor between 1/10 and 10

Tour_du_Valat ~ dnorm(0.0, 0.75) # a priori new method can increase/decrease detection proba by a factor between 1/10 and 10

Tour_du_Valat_new_counting_method ~ dnorm(0.0, 0.75) # a priori new method can increase/decrease detection proba by a factor between 1/10 and 10

Obs_1 <- 0.0

Obs_2 ~ dnorm(0.0, 1.55) # a priori Obs 2 has a detection proba between 1/5 and 5 of that of TAM

Obs_3 ~ dnorm(0.0, 1.55) # a priori Obs 3 has a detection proba between 1/5 and 5 of that of TAM

for(s in 1:n_site) {

for(t in 1:27) { gamma[1, s, t] <- Obs_1 }

for(t in 28:37) { gamma[1, s, t] <- Obs_2 }

for(t in 38:n_year) { gamma[1, s, t] <- Obs_3 }

}

for(s in 1:7) {

for(t in 1:n_year) { gamma[2, s, t] <- Marais_de_la_Palissade }

}

for(s in 8:28) {

for(t in 1:n_year) { gamma[2, s, t] <- Réserve_Naturelle_Nationale_de_Camargue }

}

for(s in 29:38) {

for(t in 1:31) { gamma[2, s, t] <- Tour_du_Valat }

for(t in 32:n_year) { gamma[2, s, t] <- Tour_du_Valat_new_counting_method }

}

for(s in 39:40) {

for(t in 1:n_year) { gamma[2, s, t] <- Réserve_Naturelle_des_Marais_du_Vigueirat }

}

}
