# Supplementary material for: Accounting for detection probability with overestimation by integrating double monitoring programs over 40 years
Source: PLoS One. 2022 Mar 25;17(3):e0265730. doi: 10.1371/journal.pone.0265730 (PMC8956176; doi:10.1371/journal.pone.0265730)
Supplement: S3 Appendix — (DOCX) [file pone.0265730.s003.docx]

**Electronic supplementary material**

**Accounting for detection probability with overestimation by integrating double monitoring programs over 40 years**

David Vallecillo^1,2*^, Matthieu Guillemain^2^, Matthieu Authier^3^, Colin Bouchard^4^, Damien Cohez^1^, Emmanuel Vialet^5^, Grégoire Massez^6^, Philippe Vandewalle^7^, Jocelyn Champagnon^1^

^1^ Tour du Valat, Research institute for the conservation of Mediterranean wetlands, Le Sambuc, 13200 Arles, France

^2^ OFB, Unité Avifaune migratrice, La Tour du Valat, Le Sambuc, 13200 Arles, France

^3^ Observatoire Pelagis, UMS 3462 CNRS-LRUniv ADERA, 17 000 La Rochelle, France

^4^ UMR Ecobiop, e2S, Université de Pau et Pays de l’Adour, INRAE, 64310 Saint-Pée sur Nivelle, France

^5^

^6^

^7^ SNPN-RNN de Camargue, 13 200 Arles, France

* Corresponding author

E-mail : [vallecillo@tourduvalat.org](mailto:vallecillo@tourduvalat.org)

**S3 Appendix**


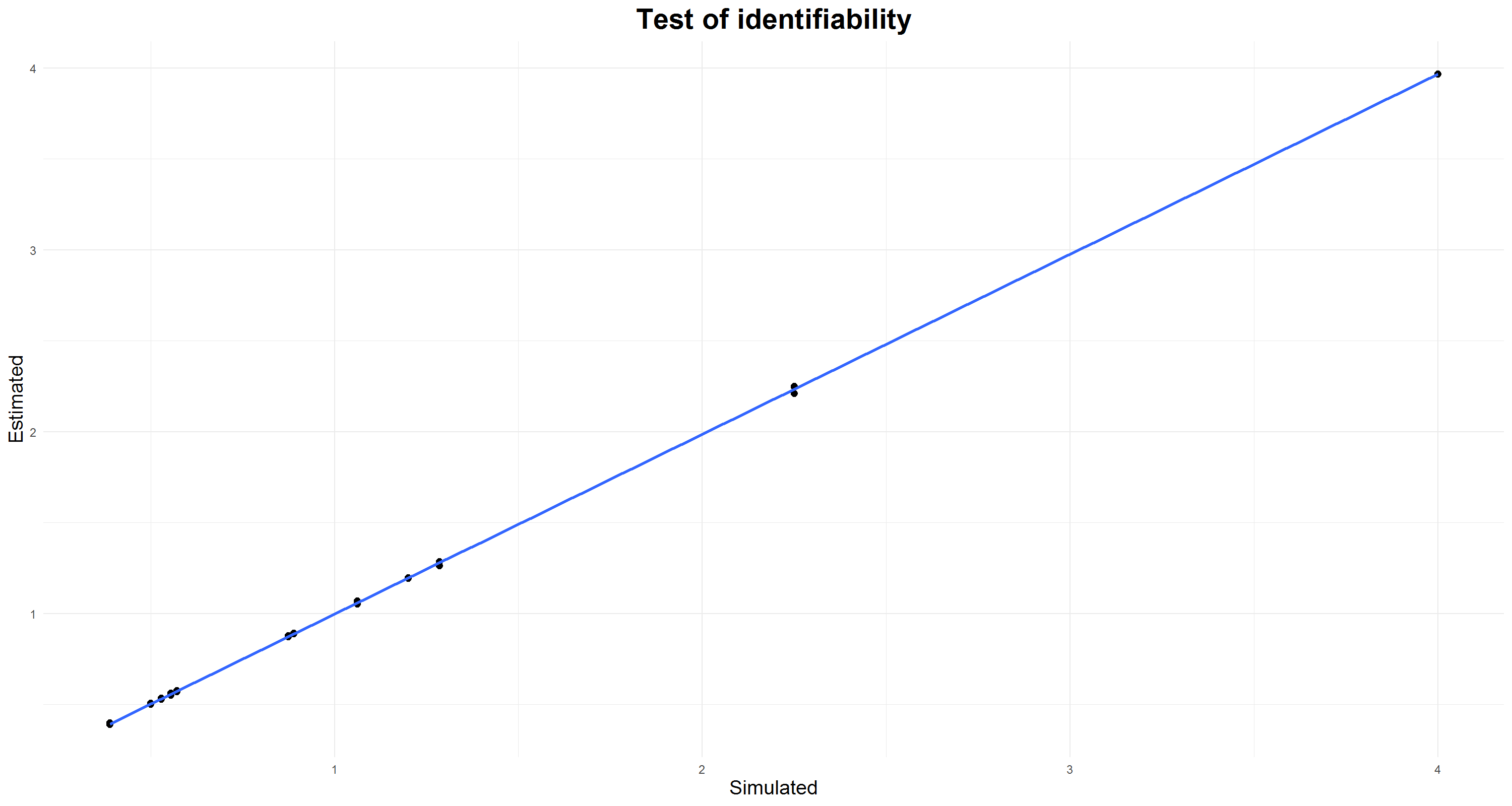


We have tested the ability of the model to estimate correctly the different parameters whatever the value set for ($\text{β}_{\text{2}}$: Réserve Naturelle des Marais du Vigueirat). First, we simulate data with the developed model by setting the parameter values. We run the model on its simulated data and then compare the output of the model to the parameters we had set to simulate the data to measure the estimation bias. The sensitivity analysis consists in setting different values for $\text{β}_{\text{2}}$ and see how it affects the other parameters.

The points of the graph represent the ratios between two conditional detection probability values for the simulated and estimated data. For a simulated data set 7 parameters are estimated and one parameter is fixed ($\text{β}_{\text{2}}$: Réserve Naturelle des Marais du Vigueirat), so 7 ratios are calculated. 5 datasets were simulated where the value of the $\text{β}_{\text{2}}$ parameter was modified as well as the detection probability values for the three observers and the three protected areas. The results of this analysis show that shifts in conditional detection probability between observers (aerial monitoring) and protected areas (ground monitoring) are estimated without bias whatever the value set for $\text{β}_{\text{2}}$.
