# Supplementary material for: Accounting for detection probability with overestimation by integrating double monitoring programs over 40 years
Source: PLoS One. 2022 Mar 25;17(3):e0265730. doi: 10.1371/journal.pone.0265730 (PMC8956176; doi:10.1371/journal.pone.0265730)
Supplement: S4 Appendix — (DOCX) [file pone.0265730.s004.docx]

**Electronic supplementary material**

**Accounting for detection probability with overestimation by integrating double monitoring programs over 40 years**

David Vallecillo^1,2*^, Matthieu Guillemain^2^, Matthieu Authier^3^, Colin Bouchard^4^, Damien Cohez^1^, Emmanuel Vialet^5^, Grégoire Massez^6^, Philippe Vandewalle^7^, Jocelyn Champagnon^1^

^1^ Tour du Valat, Research institute for the conservation of Mediterranean wetlands, Le Sambuc, 13200 Arles, France

^2^ OFB, Unité Avifaune migratrice, La Tour du Valat, Le Sambuc, 13200 Arles, France

^3^ Observatoire Pelagis, UMS 3462 CNRS-LRUniv ADERA, 17 000 La Rochelle, France

^4^ UMR Ecobiop, e2S, Université de Pau et Pays de l’Adour, INRAE, 64310 Saint-Pée sur Nivelle, France

^5^ Parc Naturel Régional de Camargue, Mas du Pont de Rousty, 13 200 Arles, France

^6^ Les Amis des Marais du Vigueirat, Chemin de l'Etourneau, 13 104 Mas-Thibert, France

^7^ SNPN-RNN de Camargue, 13 200 Arles, France

* Corresponding author

E-mail : [vallecillo@tourduvalat.org](mailto:vallecillo@tourduvalat.org)

**S4 Appendix**

**Bayesian fit analysis**


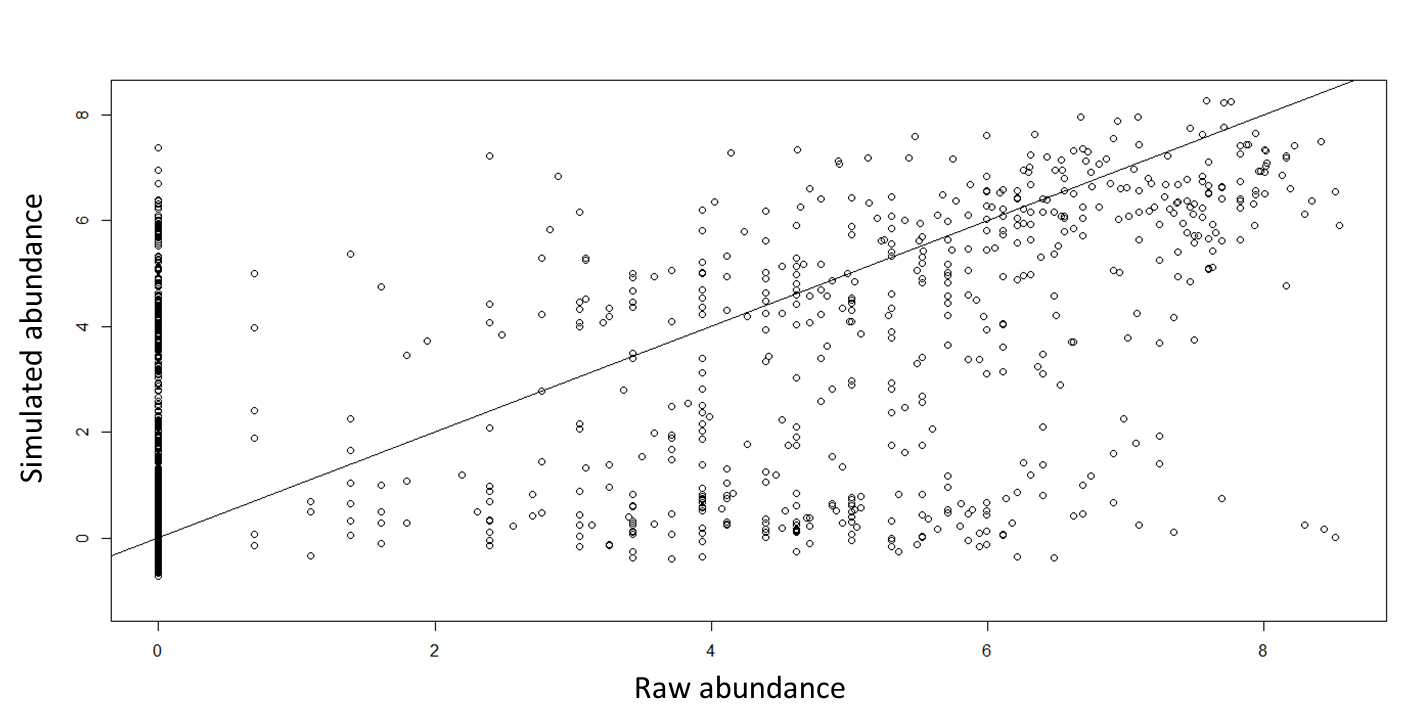


We tested the fit of the model to the observed data by plotting simulated data (using model parameter estimates) against raw data. The scales are in log and results are presented for the month of September.

The figure show the model fits the data well when individuals are present. However, it is not perfectly adapted to presence/absence data. On several occasions, the model estimates abundance when there are no individuals on site. However, this represents only a small proportion of the zeros, as in 82% of cases the model estimates zero abundance when no individuals have been counted.

This lack of fit is not a problem for the purpose of the study, as the simulation exercises showed that the conditional detection probability parameters were correctly estimated.
